# Cardiovascular–Kidney–Metabolic Overlaps, Clinical Outcomes, and Medications Among Patients With Heart Failure: Results From the Chinese Cardiovascular Association Database–Heart Failure Center Registry

**DOI:** 10.1002/mco2.71008

**Published:** 2026-09-23

**Authors:** Xiang Wang, Jingkuo Li, Yi Li, Lubi Lei, Wei Wang, Xuyang Meng, Chenxi Xia, Chenguang Yang, Lihua Zhang, Yuhua Liao, Weimin Li, Yugang Dong, Xinli Li, Jingmin Zhou, Jiefu Yang, Fang Wang

**Affiliations:** ^1^ Department of Cardiology Beijing Hospital National Center of Gerontology Institute of Geriatric Medicine Chinese Academy of Medical Sciences Beijing China; ^2^ National Clinical Research Center for Cardiovascular Diseases Fuwai Hospital National Center for Cardiovascular Diseases State Key Laboratory of Cardiovascular Disease Chinese Academy of Medical Sciences and Peking Union Medical College Beijing China; ^3^ Center for Clinical and Epidemiologic Research Beijing Anzhen Hospital Beijing Institute of Heart, Lung and Blood Vessel Diseases Capital Medical University Beijing China; ^4^ Union Hospital Tongji Medical College Huazhong University of Science and Technology Wuhan China; ^5^ The First Affiliated Hospital of Harbin Medical University Harbin China; ^6^ The First Affiliated Hospital of Sun Yat‐sen University Guangzhou China; ^7^ The First Affiliated Hospital of Nanjing Medical University Nanjing China; ^8^ Department of Cardiology Shanghai Institute of Cardiovascular Diseases, Zhongshan Hospital Fudan University Shanghai China

**Keywords:** cardiovascular–kidney–metabolic health, death, heart failure, medication, rehospitalization

## Abstract

Cardiovascular–kidney–metabolic (CKM) multimorbidity is common among patients with heart failure (HF), but its prognostic impact in Chinese patients remains largely unclear. We examined the relationship of CKM multimorbidity with adverse outcomes and its interaction with guideline‐directed medical therapy (GDMT) in patients with HF. We classified hospitalized patients with HF by CKM‐condition count (atherosclerotic cardiovascular disease [ASCVD], chronic kidney disease [CKD], and type 2 diabetes [T2D]). Cox models assessed 1‐year all‐cause death, cardiovascular (CV) death, and rehospitalizations. Among patients with HF with reduced ejection fraction (HFrEF), subgroup analyses were conducted by GDMT use. Among 126,524 patients, 66.4% had at least one CKM condition and 5.1% had all three. All‐cause death increased with CKM burden; three conditions conferred the highest risk (adjusted hazard ratio 2.43, 95% confidence interval [CI] 2.20–2.69) versus no CKM conditions. Associations were stronger in HFrEF. Although GDMT recipients had lower absolute death rates, associations of CKM multimorbidity with all outcomes were generally consistent regardless of GDMT use. CKM multimorbidity is common in Chinese patients hospitalized with HF and is associated with a worse prognosis. Although GDMT lowered absolute death rates, it did not substantially modify the relative associations between CKM multimorbidity and subsequent clinical outcomes.

## Graphical abstract

In a nationwide heart failure cohort, cardiovascular–kidney–metabolic (CKM) multimorbidity was common in Chinese hospitalized heart failure (HF) patients and associated with poor prognosis. Higher CKM burden and combinations identified high‐risk patients. Although guideline‐directed medical therapy (GDMT) reduced mortality, it did not modify these associations. Findings emphasize integrated CKM risk stratification and personalized HF management.

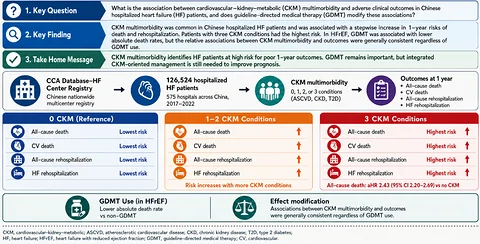

## 1 Introduction

Heart failure (HF) imposes a substantial clinical and public health burden, with high morbidity, recurrent hospitalization, impaired quality of life, and considerable mortality [1]. Patients with HF often present with cardiovascular and non‐cardiovascular comorbidities, which complicate clinical management and worsen prognosis [2, 3, 4]. Among these comorbidities, cardiovascular disease, kidney dysfunction, and metabolic disorders share risk factors and interact through bidirectional biological pathways. The term cardiovascular–kidney–metabolic (CKM) multimorbidity has therefore been increasingly recognized as an important clinical construct that reflects the overlap of atherosclerotic cardiovascular disease (ASCVD), chronic kidney disease (CKD), and type 2 diabetes (T2D) [1]. These conditions can accelerate HF through endothelial injury, inflammation, neurohormonal and metabolic abnormalities, volume expansion, and impaired renal function. The American Heart Association has proposed a CKM framework that provides definitions, stages, and risk‐prediction approaches [5], whereas contemporary HF guidelines recommend coordinated management of related comorbidities [6].

Despite increasing awareness of CKM multimorbidity, its overlap patterns and prognostic implications in HF are not fully characterized. Secondary analyses of randomized clinical trial datasets have explored whether CKM multimorbidity is associated with subsequent clinical outcomes [2, 3]. However, trial populations are often highly selected because of strict eligibility criteria, safety considerations, and treatment‐related exclusions, which may lead to under‐representation of patients with complex comorbidities [7]. In addition, most available evidence has been derived from the United States and Western Europe. Patients from low‐ and middle‐income countries (LMICs), including China, have been less frequently studied, although they may have different comorbidity profiles, healthcare access, treatment patterns, and outcome risks. Prior evidence suggests that patients from LMICs may report fewer comorbidities but experience higher mortality than those from high‐income regions [8]. Therefore, the prevalence, combinations, and prognostic significance of CKM multimorbidity observed in Western trial settings may not be directly generalizable to Chinese patients with HF.

Guideline‐directed medical therapy (GDMT) is a cornerstone of HF management, and it has been consistently associated with improved prognosis. However, implementation of GDMT in real‐world practice remains suboptimal, particularly among patients with multiple comorbidities [9]. Patients with CKM multimorbidity may be less likely to receive recommended therapies because of concerns about renal function, hypotension, hyperkalemia, drug intolerance, polypharmacy, or potential adverse effects [10, 11]. Moreover, CKM multimorbidity may mark a subgroup with high absolute risk and considerable potential benefit from treatment optimization. However, it remains unclear whether its association with adverse outcomes varies according to overall GDMT or the use of specific medications. This evidence gap is clinically important because it may help clarify whether CKM‐related risk persists across treatment strata and whether patients with CKM multimorbidity require more intensive medication optimization and multidisciplinary care.

By using the Chinese Cardiovascular Association (CCA) Database–HF Center Registry, we aimed to assess the associations of the number and combination patterns of CKM conditions with all‐cause death, cardiovascular death, all‐cause rehospitalization, and HF‐related rehospitalization among hospitalized patients with HF in China. We also evaluated whether these relationships varied according to GDMT and the use of specific medications. By jointly characterizing CKM burden, multimorbidity patterns, prognosis, and treatment‐related heterogeneity in a large real‐world HF cohort, this study may inform integrated CKM‐based risk stratification and management strategies in China.

## 2 Results

### 2.1 Baseline Characteristics

The analytic cohort comprised 126,524 patients hospitalized primarily for acute decompensation of chronic HF. Their mean age was 68.2 ± 13.5 years, and 79,831 (63.1%) were men. Baseline characteristics stratified by CKM conditions are presented in Table 1. At least one CKM condition was present in 84,052 patients (66.4%), whereas 6487 (5.1%) had all three conditions. Increasing CKM burden was associated with older age, higher body mass index (BMI), and a greater prevalence of hypertension. In contrast, angiotensin‐converting enzyme inhibitors/angiotensin receptor blocker (ACEI/ARB) and mineralocorticoid receptor antagonist (MRA) use at discharge was less frequent among patients with more CKM conditions.

**TABLE 1:** Baseline characteristics by numbers of cardiovascular–kidney–metabolic (CKM) conditions.

Variables	Total (*n* = 126,524)	0 (*n* = 42,472)	1 (*n* = 49,891)	2 (*n* = 27,674)	3 (*n* = 6487)	*p* value
Age, years (mean [SD])	68.20 (13.54)	65.40 (14.99)	68.88 (13.02)	70.43 (11.85)	71.90 (10.66)	<0.001
Male, *n* (%)	79,831 (63.1)	26,566 (62.5)	32,251 (64.6)	17,247 (62.3)	3767 (58.1)	<0.001
BMI, kg/m^2^ (mean [SD])	24.07 (4.44)	23.93 (4.66)	24.04 (4.43)	24.27 (4.21)	24.38 (3.98)	<0.001
SBP, mmHg (mean [SD])	126.20 (21.62)	125.00 (21.35)	125.98 (21.58)	127.59 (21.89)	129.80 (21.87)	<0.001
Heart rate, bpm (mean [SD])	79.40 (17.78)	81.62 (19.45)	78.68 (17.29)	77.92 (16.07)	76.77 (15.17)	<0.001
NT‐proBNP, pg/mL, (mean [SD])	653.42 (1569.27)	605.11 (1543.72)	610.73 (1464.98)	734.55 (1702.25)	951.81 (1848.31)	<0.001
LVEF phenotypes, *n* (%)						<0.001
HFrEF	44,720 (35.3)	17,513 (41.2)	16,773 (33.6)	8537 (30.8)	1897 (29.2)	
HFmrEF	31,802 (25.1)	9302 (21.9)	12,955 (26.0)	7739 (28.0)	1806 (27.8)	
HFpEF	50,002 (39.5)	15,657 (36.9)	20,163 (40.4)	11,398 (41.2)	2784 (42.9)	
NYHA class, *n* (%)						<0.001
I	33,253 (26.3)	11,515 (27.1)	13,718 (27.5)	6778 (24.5)	1242 (19.1)	
II	38,885 (30.7)	11,593 (27.3)	15,537 (31.1)	9363 (33.8)	2392 (36.9)	
III	34,606 (27.4)	12,403 (29.2)	13,278 (26.6)	7183 (26.0)	1742 (26.9)	
IV	19,780 (15.6)	6961 (16.4)	7358 (14.7)	4350 (15.7)	1111 (17.1)	
Pulmonary rale, *n* (%)	34,022 (26.9)	11,546 (27.2)	13,085 (26.2)	7526 (27.2)	1865 (28.7)	<0.001
Peripheral edema, *n* (%)	26,849 (21.2)	9823 (23.1)	9900 (19.8)	5681 (20.5)	1445 (22.3)	<0.001
Jugular vein distension, *n* (%)	13,679 (10.8)	5222 (12.3)	4991 (10.0)	2781 (10.0)	685 (10.6)	<0.001
Hypertension, *n* (%)	75,545 (59.7)	20,176 (47.5)	30,323 (60.8)	19,745 (71.3)	5301 (81.7)	<0.001
Type 2 diabetes, *n* (%)	35,855 (28.3)	0 (0.0)	11,056 (22.2)	18,807 (68.0)	5992 (92.4)	<0.001
Stroke, *n* (%)	14,963 (11.8)	0 (0.0)	6822 (13.7)	6117 (22.1)	2024 (31.2)	<0.001
MI, *n* (%)	33,442 (26.4)	0 (0.0)	16,150 (32.4)	13,448 (48.6)	3844 (59.3)	<0.001
PCI, *n* (%)	18,214 (14.4)	0 (0.0)	9716 (19.5)	6920 (25.0)	1578 (24.3)	<0.001
CABG, *n* (%)	1226 (1.0)	0 (0.0)	557 (1.1)	513 (1.9)	156 (2.4)	<0.001
Peripheral artery disease, *n* (%)	12,228 (9.7)	0 (0.0)	5405 (10.8)	4967 (17.9)	1856 (28.6)	<0.001
CKD, *n* (%)	16,324 (12.9)	0 (0.0)	4833 (9.7)	7239 (26.2)	4252 (65.5)	<0.001
AF, *n* (%)	37,604 (29.7)	14,927 (35.1)	14,244 (28.6)	6939 (25.1)	1494 (23.0)	<0.001
COPD, *n* (%)	11,488 (9.1)	4135 (9.7)	4555 (9.1)	2286 (8.3)	512 (7.9)	<0.001
Anemia, *n* (%)	643 (0.5)	98 (0.2)	238 (0.5)	217 (0.8)	90 (1.4)	<0.001
ACEI/ARB, *n* (%)	59,441 (47.0)	20,538 (48.4)	24,296 (48.7)	12,275 (44.4)	2332 (35.9)	<0.001
ARNI, *n* (%)	43,647 (34.5)	14,885 (35.0)	16,658 (33.4)	9729 (35.2)	2375 (36.6)	<0.001
Beta‐receptor blocker, *n* (%)	102,072 (80.7)	34,163 (80.4)	40,318 (80.8)	22,383 (80.9)	5208 (80.3)	0.315
MRA, *n* (%)	94,130 (74.4)	33,964 (80.0)	36,496 (73.2)	19,445 (70.3)	4225 (65.1)	<0.001
SGLT2i, *n* (%)	4542 (3.6)	1385 (3.3)	1771 (3.5)	1110 (4.0)	276 (4.3)	<0.001

Abbreviations: ACEI, angiotensin converting enzyme inhibitors; AF, atrial fibrillation; ARB, angiotensin receptor blocker; ARNI, angiotensin receptor neprilysin inhibitor; BMI, body mass index; CABG, coronary artery bypass grafting; CKD, chronic kidney disease; COPD, chronic obstructive pulmonary disease; HFmrEF, HF with a mildly reduced fraction; HFpEF, HF with preserved ejection fraction; HFrEF, HF with reduced ejection fraction; LVEF, left ventricular ejection fraction; MI, myocardial infarction; MRA, mineralocorticoid receptor antagonist; NT‐proBNP, N‐terminal pro‐brain natriuretic peptide; NYHA, New York Heart Association; PCI, percutaneous coronary intervention; SBP, systolic blood pressure; SD, standard deviation; SGLT2i, sodium‐glucose cotransporter 2 inhibitors.

Across specific patterns, 27,387 (21.6%) had ASCVD alone, 9966 (7.9%) CKD alone, 12,538 (9.9%) T2D alone, 7176 (5.7%) ASCVD + CKD, 5387 (4.3%) CKD + T2D, and 15,111 (11.9%) ASCVD + T2D (Table S1). CKD‐containing patterns were associated with older age, higher N‐terminal pro‐brain natriuretic peptide (NT‐proBNP), and more hypertension. T2D‐containing patterns showed higher BMI and systolic blood pressure (SBP) and less chronic obstructive pulmonary disease (COPD), whereas patients with ASCVD in their pattern were more often male and had more pulmonary rales. Tables S2 and S3 provide characteristics according to NT‐proBNP availability and left ventricular ejection fraction (LVEF) phenotype, respectively.

### 2.2 Associations of CKM With 1‐Year Clinical Outcomes

**FIGURE 1:**
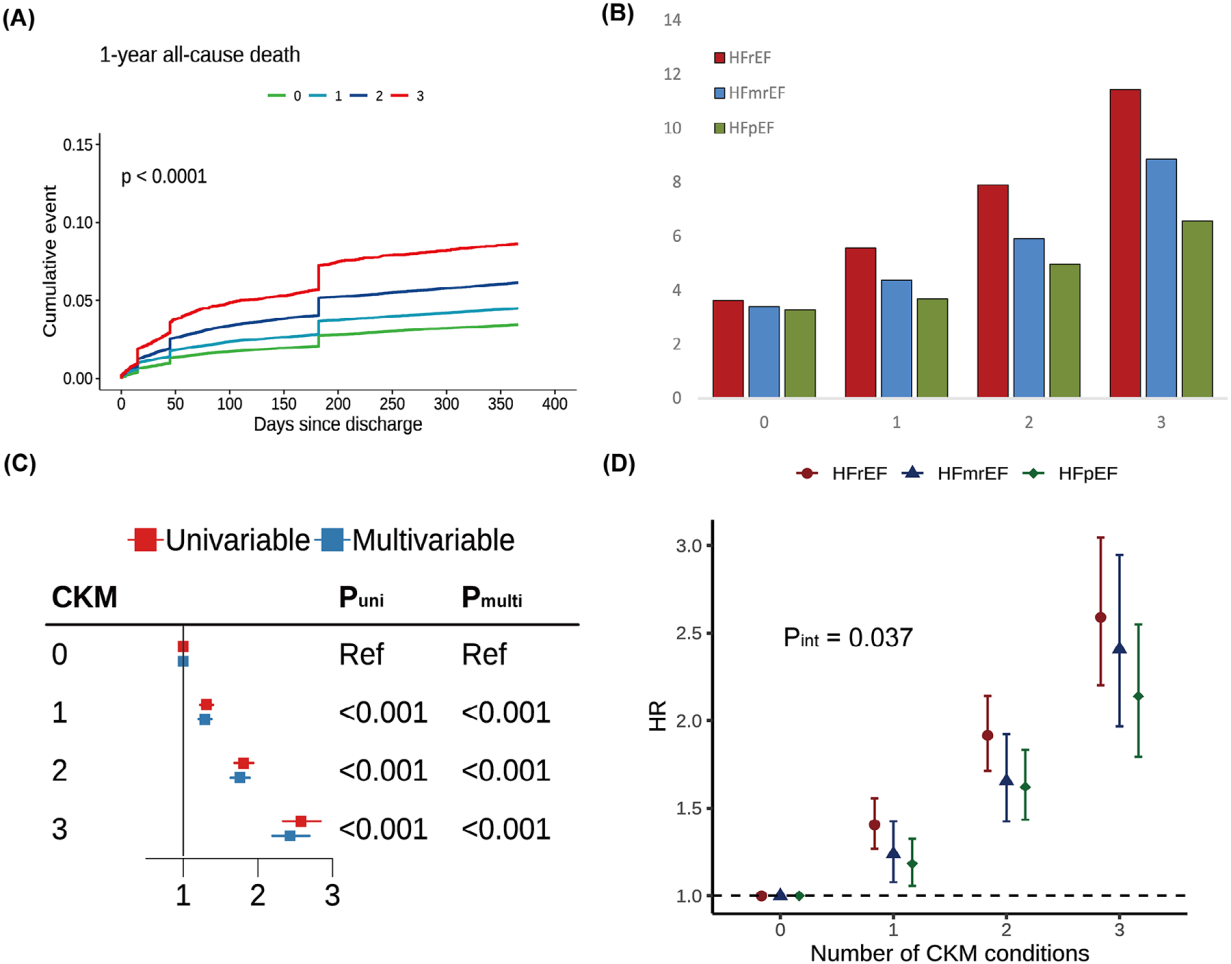
1‐year all‐cause death risk according to numbers of CKM conditions and LVEF phenotypes. (A) Kaplan–Meier curves showing 1‐year all‐cause death stratified by numbers of CKM conditions. (B) Absolute 1‐year all‐cause death risk by numbers of CKM conditions and LVEF phenotypes. (C) Univariable and multivariable analysis of all‐cause death risk according to numbers of CKM conditions. The reference (Ref) is not CKM (D) multivariable analysis of all‐cause death risk by numbers of CKM conditions and LVEF phenotypes. Multivariable analysis was adjusted for age, sex, BMI, SBP, heart rate, NYHA class, NT‐proBNP, LVEF, hypertension, AF, COPD, anemia, and medications prescribed at discharge (ACEI/ARB, ARNI, beta‐receptor blocker, MRA, SGLT‐2i). CKM, cardiovascular–kidney–metabolic disease; HFmrEF, HF with a mildly reduced fraction; HFpEF, HF with preserved ejection fraction; HFrEF, HF with reduced ejection fraction; HR, hazard ratio.

**FIGURE 2:**
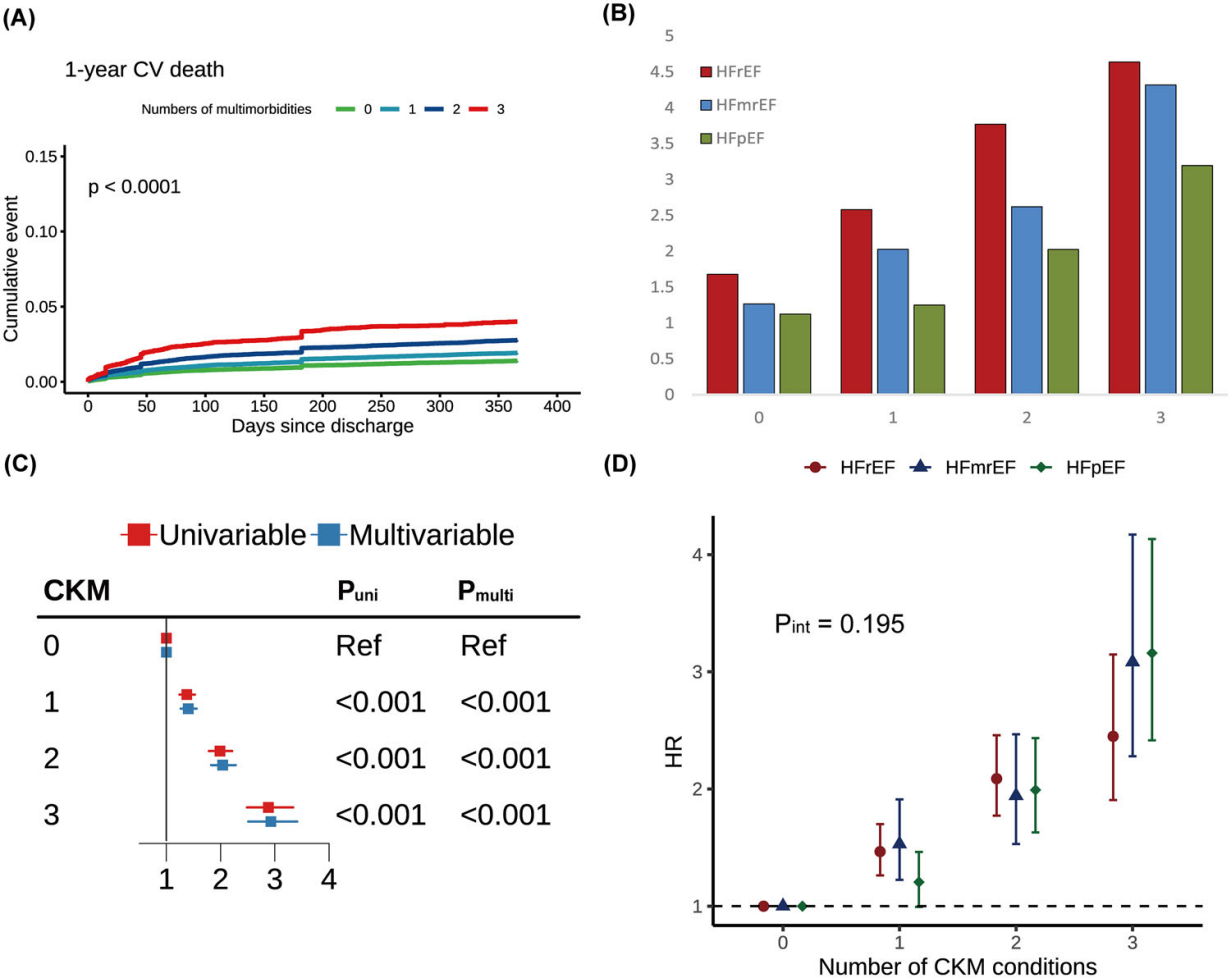
1‐year CV death risk according to numbers of CKM conditions and LVEF phenotypes. (A) Kaplan–Meier curves showing 1‐year CV death stratified by numbers of CKM conditions. (B) Absolute 1‐year CV death risk by numbers of CKM conditions and LVEF phenotypes. (C) Univariable and multivariable analysis of CV death risk according to numbers of CKM conditions. The reference (Ref) is no CKM (D) multivariable analysis of CV death risk by numbers of CKM conditions and LVEF phenotypes. Multivariable analysis was adjusted for age, sex, BMI, SBP, heart rate, NYHA class, NT‐proBNP, LVEF, hypertension, AF, COPD, anemia, and medications prescribed at discharge (ACEI/ARB, ARNI, beta‐receptor blocker, MRA, SGLT‐2i). CKM, cardiovascular–kidney–metabolic disease; HFmrEF, HF with a mildly reduced fraction; HFpEF, HF with preserved ejection fraction; HFrEF, HF with reduced ejection fraction; HR, hazard ratio.

**FIGURE 3:**
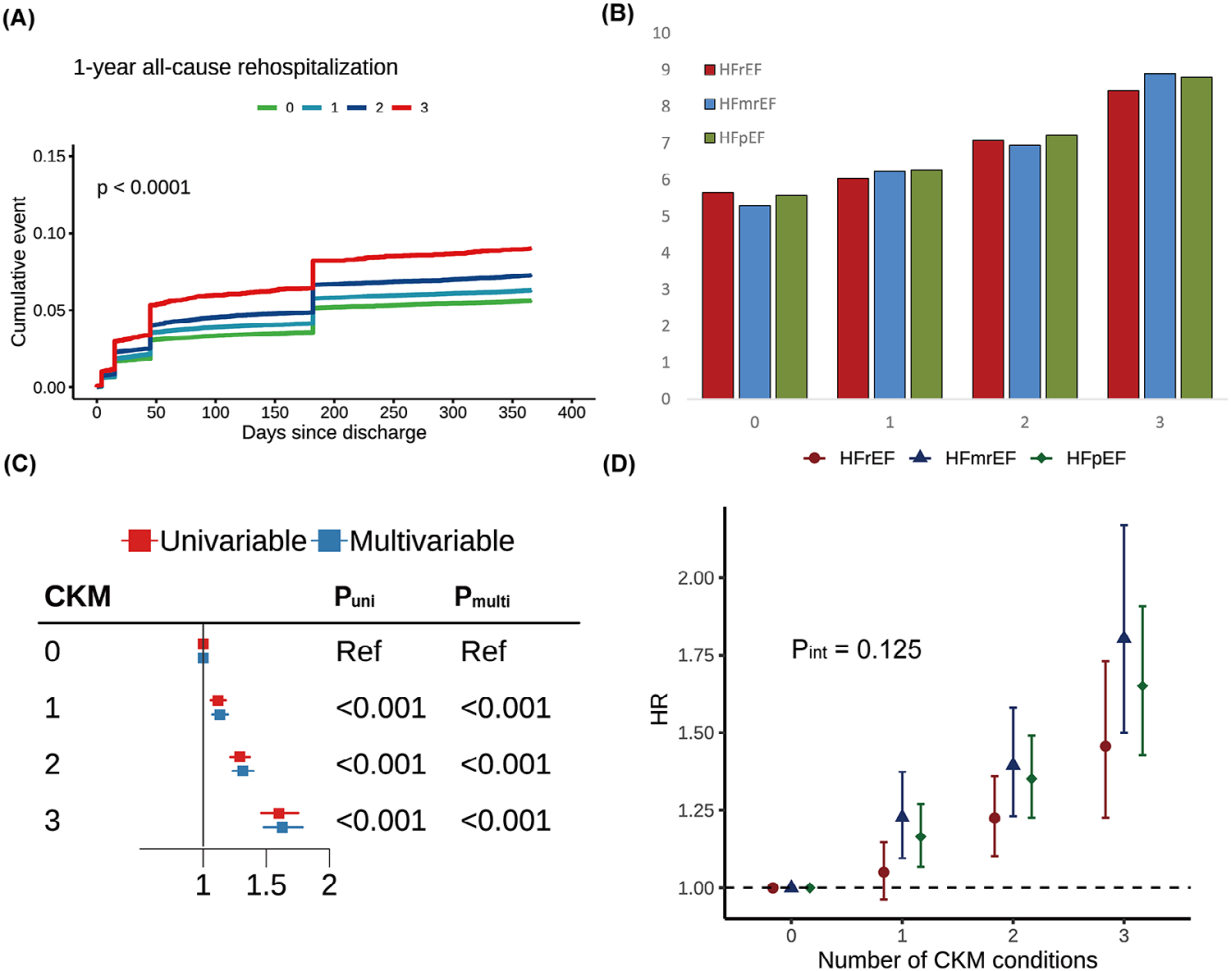
1‐year all‐cause rehospitalization risk according to numbers of CKM conditions and LVEF phenotypes. (A) Kaplan–Meier curves showing 1‐year all‐cause rehospitalization stratified by numbers of CKM conditions. (B) Absolute 1‐year all‐cause rehospitalization risk by numbers of CKM conditions and LVEF phenotypes. (C) Univariable and multivariable analysis of all‐cause rehospitalization risk according to numbers of CKM conditions. The reference (Ref) is not CKM (D) multivariable analysis of all‐cause rehospitalization risk by numbers of CKM conditions and LVEF phenotypes. Multivariable analysis was adjusted for age, sex, BMI, SBP, heart rate, NYHA class, NT‐proBNP, LVEF, hypertension, AF, COPD, anemia, and medications prescribed at discharge (ACEI/ARB, ARNI, beta‐receptor blocker, MRA, SGLT‐2i). CKM, cardiovascular–kidney–metabolic disease; HFmrEF, HF with a mildly reduced fraction; HFpEF, HF with preserved ejection fraction; HFrEF, HF with reduced ejection fraction; HR, hazard ratio.

**FIGURE 4:**
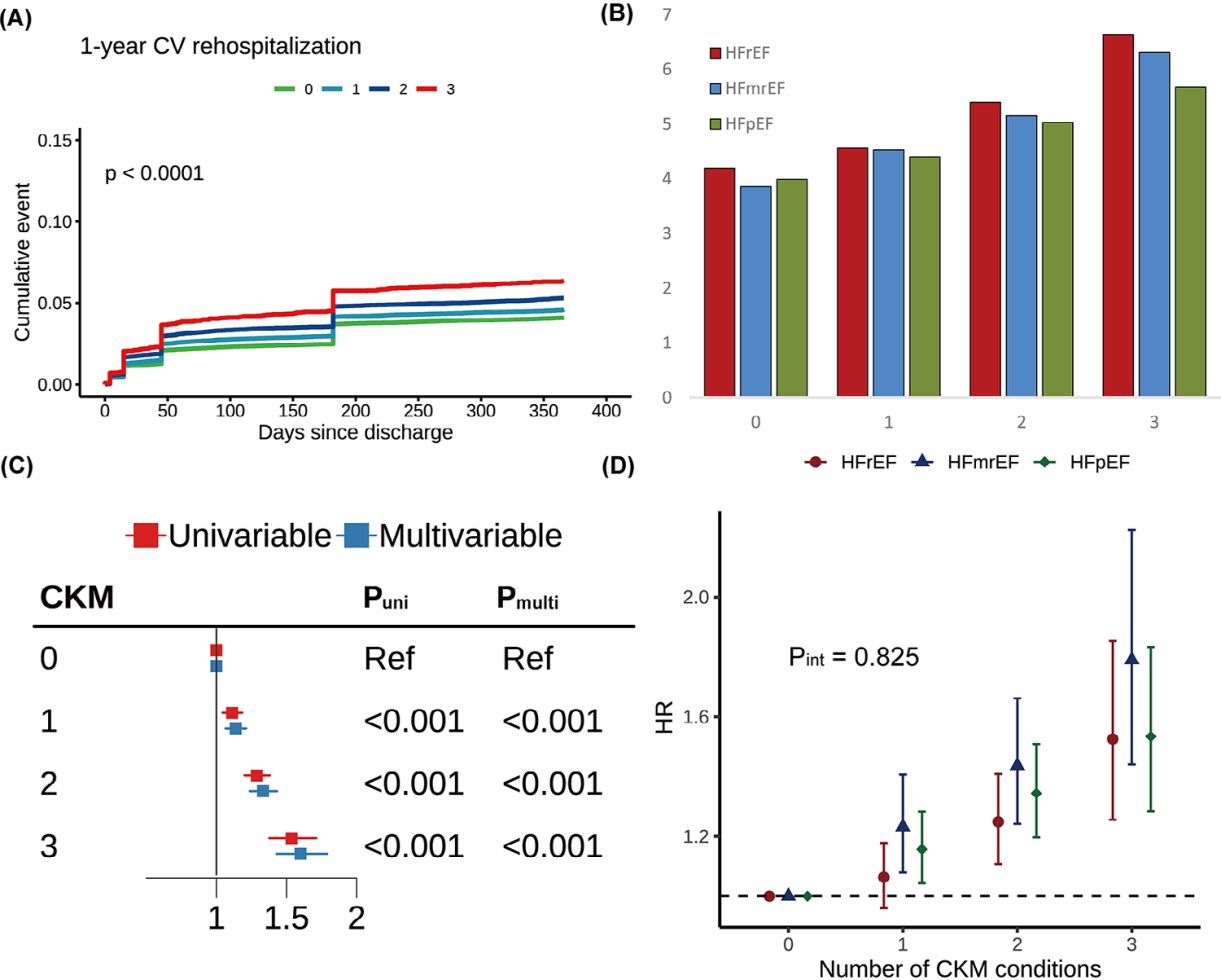
1‐year CV rehospitalization risk according to numbers of CKM conditions and LVEF phenotypes. (A) Kaplan–Meier curves showing 1‐year CV rehospitalization stratified by numbers of CKM conditions. (B) Absolute 1‐year CV rehospitalization risk by numbers of CKM conditions and LVEF phenotypes. (C) Univariable and multivariable analysis of CV rehospitalization risk according to numbers of CKM conditions. The reference (Ref) is not CKM (D) multivariable analysis of CV rehospitalization risk by numbers of CKM conditions and LVEF phenotypes. Multivariable analysis was adjusted for age, sex, BMI, SBP, heart rate, NYHA class, NT‐proBNP, LVEF, hypertension, AF, COPD, anemia, and medications prescribed at discharge (ACEI/ARB, ARNI, beta‐receptor blocker, MRA, SGLT‐2i). CKM, cardiovascular–kidney–metabolic disease; HFmrEF, HF with a mildly reduced fraction; HFpEF, HF with preserved ejection fraction; HFrEF, HF with reduced ejection fraction; HR, hazard ratio.

During 1 year of follow‐up, 5961 of 126,524 patients died (4.7%), and 2547 of these deaths (42.7%) were attributed to CV causes. Rehospitalization occurred in 7974 patients (6.3%), including 5790 patients (72.6%) with CV rehospitalization. All‐cause mortality increased steadily with the accumulation of CKM conditions; 1‐year all‐cause death increased from 3.4% among patients without CKM conditions to 8.3% among those with three CKM conditions (Figure 1A). Similar patterns were seen across LVEF phenotypes, with the highest mortality observed in patients with HF with reduced ejection fraction (HFrEF) (Figure 1B). In the fully adjusted analysis, the presence of all three CKM conditions was associated with a significantly elevated risk of all‐cause mortality compared with having none (adjusted hazard ratio [aHR] 2.43, 95% confidence interval [CI] 2.20–2.69) (Figure 1C). The association between CKM condition number and all‐cause death was stronger in patients with HFrEF (*p* for interaction = 0.037) (Figure 1D). Similar results of CV death, all‐cause rehospitalization, and CV rehospitalization are shown in Figures 2, 3, 4, respectively. However, for these three outcomes, the association with CKM condition number did not differ significantly across LVEF phenotypes (all *p* for interaction >0.05). Additional analyses by CKD stage yielded broadly similar associations between CKM burden and each clinical outcome, with none of the interaction tests reaching statistical significance (all *p* for interaction >0.05) (Tables S4).

**FIGURE 5:**
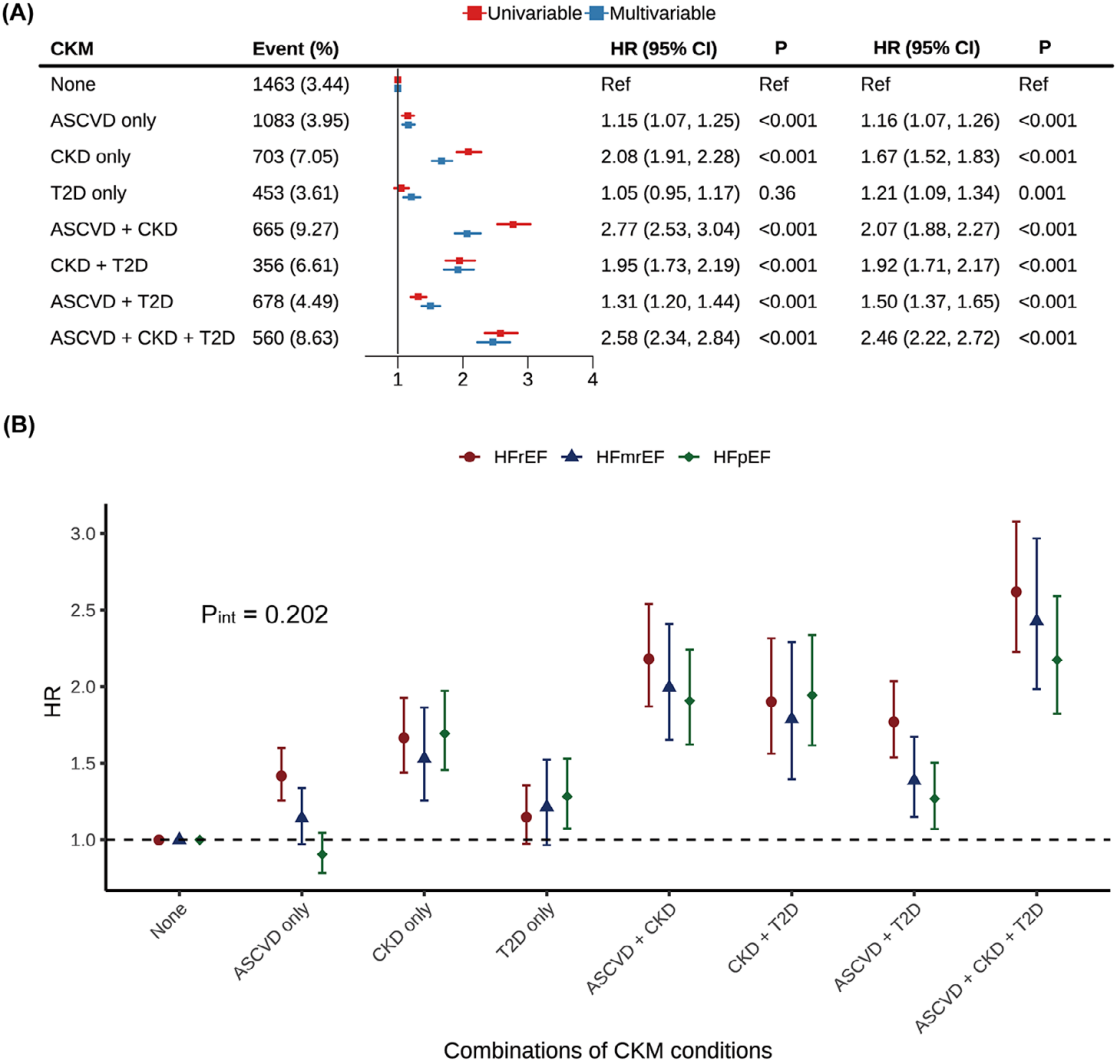
1‐year all‐cause death risk according to combinations of CKM conditions and LVEF phenotypes. (A) Univariable and multivariable analysis of all‐cause death risk according to combinations of CKM conditions. The reference (Ref) is not CKM (B) multivariable analysis of all‐cause death risk by combinations of CKM conditions and LVEF phenotypes. Multivariable analysis was adjusted for age, sex, BMI, SBP, heart rate, NYHA class, NT‐proBNP, LVEF, hypertension, AF, COPD, anemia, and medications prescribed at discharge (ACEI/ARB, ARNI, beta‐receptor blocker, MRA, SGLT‐2i). ASCVD, atherosclerotic cardiovascular disease; CI, confidence interval; CKD, chronic kidney disease; CKM, cardiovascular–kidney–metabolic disease; HR, hazard ratio; Ref, reference; T2D, type 2 diabetes.

Outcome risk also differed by the specific CKM pattern. For one‐condition patterns, CKD alone had the highest all‐cause death risk (aHR 1.67, 95% CI 1.52–1.83), followed by T2D alone (aHR 1.21, 95% CI 1.09–1.34) and ASCVD alone (aHR 1.16, 95% CI 1.07–1.26). Among two‐condition patterns, ASCVD + CKD and CKD + T2D were associated with higher risk (aHR 2.07, 95% CI 1.88–2.27; and aHR 1.92, 95% CI 1.71–2.17, respectively) than ASCVD + T2D (aHR 1.50, 95% CI 1.37–1.65; Figure 5A). Results for the other outcomes are shown in Figures S1A, S2A, and S3A. Associations did not materially differ by LVEF phenotype (Figure 5B; Figures S1B, S2B, and S3B) or CKD stage (all *p* for interaction >0.05; Table S5).

Excluding deaths within 30 days of discharge attenuated the associations but did not change their direction (Table S6). After applying inverse propensity score weighting to address missing NT‐proBNP data, outcome rates across CKM condition groups and the corresponding associations were not materially different from the main results (Table S6). Analyses based on CKM combination patterns produced similar findings (Table S7).

### 2.3 Effects of CKM on Clinical Outcomes in Patients Who Had Medications at Discharge and Who Had Not

**FIGURE 6:**
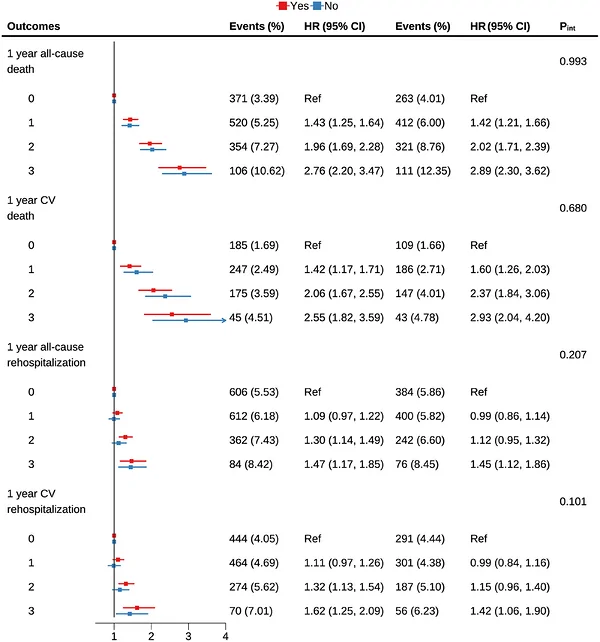
1‐year risk of death and rehospitalization according to numbers of CKM conditions and GDMT treatment in patients with HFrEF. Models were adjusted for age, sex, BMI, SBP, heart rate, NYHA class, NT‐proBNP, LVEF, hypertension, AF, COPD, and anemia. CI, confidence interval; CV, cardiovascular; HR, hazard ratio; Ref, reference.

In HFrEF, GDMT use at discharge was less frequent as CKM burden increased: 62.6% without CKM conditions versus 52.6% with all three (Table S8 and Figure S4A). Mortality from any cause and CV death were lower among GDMT recipients. However, the relationship between CKM count and death or rehospitalization did not differ by GDMT status (all *p* for interaction >0.05; Figure 6). Subgroup analyses for ACEI/ARB, angiotensin receptor neprilysin inhibitor (ARNI), beta‐receptor blocker, MRA, and sodium‐glucose cotransporter 2 inhibitors (SGLT‐2i) are presented in Figures S5–S9. The CKM‐related association with CV rehospitalization was weaker among patients receiving MRA (*p* for interaction = 0.042; Figure S8).

GDMT use also varied by CKM pattern. Prescription was lowest when CKD was present alone or with another condition (CKD only 46.7%, ASCVD + CKD 48.8%, CKD + T2D 48.3%, and all three 52.6%); the highest proportion was observed for ASCVD + T2D (63.9%; Table S9 and Figure S10A). For HFrEF, combination‐pattern associations with death and rehospitalization were not modified by GDMT status (all *p* for interaction >0.05; Figure S11). Results for individual drug classes are shown in Figures S12–S16.

## 3 Discussion

In this analysis of the CCA Database–HF Center Registry, we provide one of the largest evaluations to date of CKM multimorbidity burden and prognosis among patients with HF across the EF spectrum. Three observations were notable. CKM multimorbidity was frequent among patients hospitalized with acute HF; its accumulation was accompanied by greater risks of death and rehospitalization, with a more pronounced mortality association in HFrEF; and, despite lower absolute mortality among patients receiving GDMT, CKM‐related relative risks were similar across GDMT groups. The same general pattern was seen for individual therapies and across the LVEF spectrum.

Population aging and shared cardiometabolic risk factors have made CKM multimorbidity increasingly relevant [12]. The prevalence of CKM multimorbidity in this registry was broadly consistent with findings from previous HF trials and registries, although reported prevalence varies across study settings. In the TOPCAT trial, 72.7% had at least one CKM condition, and 34.2% had more than two [3]. DELIVER, FINEARTS‐HF, and PARAGON‐HF reported even higher CKM multimorbidity prevalence rates of 83.0%, 83.4%, and 84.4%, respectively, indicating that more than four‐fifths of trial participants had at least one condition beyond HF [3]. The Swedish HF Registry reported that 94% of patients had at least one cardiovascular comorbidity [13]. Our findings are consistent with previous evidence showing a lower multimorbidity burden in Northeast Asia than in Euro‐American regions [14]. Limited healthcare resources and underdiagnosis may partly explain the lower observed prevalence of CKM multimorbidity in China [15]. Nonetheless, the frequency of CKM multimorbidity underscores the multisystem nature of HF and the associated burden of morbidity, premature death, and healthcare use, supporting earlier detection and active management.

Greater cumulative CKM burden was strongly associated with mortality and rehospitalization, consistent with prior studies in patients with HF with a mildly reduced fraction (HFmrEF) or HF with preserved ejection fraction (HFpEF) [2, 3, 4]. The association between CKM burden and death was stronger, and the absolute death rate was higher, in patients with HFrEF than in those with HFmrEF or HFpEF. These results suggest that CKM multimorbidity is an important determinant of mortality in HFrEF. Patients with HFrEF and CKM multimorbidity also appeared to be undertreated. Previous studies have shown lower ACEI/ARB use among patients with HFrEF and high comorbidity burden [14, 16]. This population may have poorer treatment adherence, lower medication tolerance, and greater susceptibility to GDMT‐related adverse effects [15, 17]. By demonstrating poorer outcomes when CKM conditions coexist, our study reinforces the importance of CKM‐oriented research and multidisciplinary care for patients with HF.

We also observed marked heterogeneity in risk across CKM combination patterns. Patients with CKD alone, or CKD combined with ASCVD or T2D, had higher risks of adverse outcomes than those with ASCVD alone, T2D alone, or ASCVD + T2D. CKD and HF are closely linked, as reflected by the cardiorenal syndrome framework [18]. In HF, reduced cardiac output may cause chronic renal hypoperfusion, whereas sodium and water retention in CKD can worsen cardiac dysfunction. CKD also complicates HF management because clinicians must balance symptom relief and prognostic treatment against the possibility of worsening kidney function [19]. Patients with both HF and CKD are frequently less likely to receive newer guideline‐recommended therapies with kidney and metabolic benefits [13, 20]. Our results suggest that clinicians should consider both the count and composition of CKM conditions. When further examining the interactions between CKD severity and CKM multimorbidity, the results suggest that the prognostic impact of CKM multimorbidity is relatively consistent across CKD stages, indicating that CKM multimorbidity burden remains a key determinant of prognosis across CKD strata and may provide incremental risk stratification beyond CKD severity alone. Identifying and treating coexisting CKM conditions in HF is important because targeted management of comorbidities and underlying phenotypes may improve clinical outcomes. Our analytic approach differs from the full AHA CKM staging framework in that HF itself was not counted as a CKM variable, given that all participants already had a confirmed diagnosis of HF at enrollment. This departure is a deliberate analytic choice to assess the incremental prognostic burden of coexisting metabolic and vascular comorbidities within an HF population, consistent with methods adopted in prior large‐scale CKM analyses in HF trials.

We observed that patients receiving GDMT generally experienced lower absolute death rates than those not receiving GDMT. However, no appreciable interaction was observed between GDMT use and CKM multimorbidity on relative risk estimates. These findings suggest that GDMT may provide prognostic benefit across the spectrum of CKM burden, whereas the relative increase in risk associated with accumulating CKM conditions remains largely preserved. Among patients with HFrEF, those receiving GDMT had lower outcome rates than those not receiving GDMT, but the associations between CKM multimorbidity and outcomes were similar across GDMT strata. Our findings also highlight the underuse of HF pharmacotherapy. GDMT implementation remained limited in patients with HFrEF, particularly among those with CKM multimorbidity [21, 22]. MRA use at discharge was more common in this cohort than reported in some other registries, potentially reflecting initiatives to improve MRA implementation in Chinese patients with HF [23]. However, prescription remained less frequent among those with concomitant CKD [10, 24], despite recent evidence indicating that MRA therapy can be used safely across the full estimated glomerular filtration rate (eGFR) spectrum [25]. Evidence from several randomized trials has consistently demonstrated cardiometabolic and renal benefits with SGLT2 inhibitor therapy. In a secondary analysis of DELIVER, dapagliflozin remained safe and effective across different CKM profiles, whereas its absolute benefit was greatest among patients presenting with all three conditions [4]. In the present study, however, fewer than 5% of patients with HF were prescribed SGLT‐2i, likely because HF indications for this therapy were approved after the enrollment period. Beyond pharmacological management, lifestyle modification and complementary approaches—including components of the E(e)SEEDi framework (Exercise, Sleep, Stress management, Education, Diet, and interpersonal relationships) and traditional Chinese medicine—may offer additional benefits for patients with HF and CKM multimorbidity [26, 27]. However, data on these factors were not collected in the current registry, and their potential roles warrant investigation in future studies. Continued efforts are needed to improve the consistency of clinical practice and the quality of HF care in China [28].

This study benefited from a nationwide, multicenter registry, a large contemporary Chinese HF cohort, and extended follow‐up. Several limitations should also be acknowledged. First, although standardized operating procedures were used across sites, regional variation in diagnostic resources and local practice standards may have affected data quality and collection. Differences between secondary and tertiary hospitals, as well as sociocultural variation, may also have influenced the reporting of comorbidities and medication prescribing. Furthermore, ASCVD in this study was ascertained based on patient‐reported and physician‐confirmed history of qualifying acute events. Patients with coronary artery disease documented only by elective coronary angiography without prior acute events were not classified as ASCVD, potentially underestimating the true prevalence of ASCVD in this cohort. Second, reverse causality cannot be fully excluded because worsening clinical status may contribute to both CKM deterioration and adverse outcomes. Although we performed a sensitivity analysis excluding patients who died within 30 days after discharge, the results should still be interpreted cautiously. Third, some baseline characteristics were incomplete because of the retrospective design, which is a common challenge in real‐world registries and may lead to biased estimates. For instance, detailed information on specific reasons for GDMT medication intolerance or contraindications was largely unavailable, precluding more granular stratification and potentially limiting the assessment of treatment patterns. Notably, patients with missing NT‐proBNP data were older, had higher BMI, and were more likely to have HFpEF or New York Heart Association (NYHA) III class, suggesting that the missingness may not be completely random. Although multiple imputation and sensitivity analyses were performed to reduce the impact of missing data, residual bias due to non‐random missingness cannot be completely ruled out. Fourth, although CKD classification was based on documented CKD history or reduced eGFR consistent with a preexisting clinical diagnosis, serial eGFR measurements over at least 3 months were unavailable in this registry; therefore, potential misclassification of transient renal dysfunction or acute kidney injury as CKD could not be completely ruled out. Fifth, our study period overlapped with the COVID‐19 pandemic in China (2020–2022). As COVID‐19 diagnosis and infection status were not captured in the registry, the potential confounding or modifying effects of COVID‐19 on clinical outcomes could not be evaluated and represent a limitation of the present study. In addition, quality‐of‐life data were not available, although this is an important patient‐centered outcome in HF in addition to death and rehospitalization.

In this Chinese HF registry, CKM multimorbidity was common, and increasing CKM burden was associated with higher risks of all‐cause and CV death and rehospitalization. GDMT was associated with lower absolute mortality, but it did not materially change the relative associations between CKM burden and outcomes. These findings support integrated approaches to identify and manage cardiovascular, renal, and metabolic comorbidities in HF.

## 4 Methods

### 4.1 Study Population

The CCA Database–HF Center Registry is a nationwide, multicenter registry approved by the Central Ethics Committee of Beijing Hospital (2023BJYYEC‐024‐04) [29]. From January 2017 through November 2022, 575 certified secondary and tertiary hospitals in all 31 provinces of mainland China contributed data. We included consecutive inpatients whose principal discharge diagnosis was HF caused by acute decompensation of chronic HF; patients admitted primarily for another condition with HF as a secondary diagnosis were excluded. As this was a retrospective observational study, the requirement for written informed consent was waived. The initial cohort comprised 126,595 patients with an evidence‐based HF diagnosis and without serious conditions at enrollment (acute myocardial infarction [MI], malignancy, renal replacement therapy, or an implanted cardiovascular electronic device) and linkage to the Chinese Center for Disease Control and Prevention National Mortality Registration Information Management System. We then excluded 50 in‐hospital deaths, 2 patients lacking death or rehospitalization information, and 19 with LVEF >80%, leaving 126,524 patients for analysis (Figure S17).

### 4.2 CKM Multimorbidity Assessment

Baseline data were collected through standardized questionnaires and physical examination, and local laboratories performed blood testing. The 2021 CKD Epidemiology Collaboration equation without race or ethnicity coefficients was used to calculate eGFR [30].

At baseline, patients were categorized according to the presence of CKM conditions. The three components were ASCVD, CKD, and T2D [31]. ASCVD required a history of MI, percutaneous coronary intervention (PCI), coronary artery bypass grafting (CABG), stroke, or peripheral artery disease documented in the medical record from patient or physician report. Coronary disease found only by elective angiography without a qualifying acute event was not captured and was not counted. CKD was defined by a prior CKD diagnosis or eGFR <60 mL/min/1.73 m^2^ at enrollment. A documented CKD history was considered to satisfy chronicity; an isolated low eGFR was counted only when consistent with a preexisting clinical diagnosis rather than solely an acute decline during hospitalization. T2D was defined by a history of T2D or HbA1c ≥6.5%; glucose‐lowering therapy was not used as a diagnostic criterion because some agents have non‐diabetes indications. HF was not included as a CKM component because it was present in all participants. Instead, we quantified the number and combination of ASCVD, CKD, and T2D, the comorbidities most relevant to HF prognosis and commonly used in prior CKM analyses. Groups were defined by CKM count (0–3) and pattern: none, ASCVD only, CKD only, T2D only, ASCVD + CKD, CKD + T2D, ASCVD + T2D, or all three.

### 4.3 Ascertainment of Outcomes

Prespecified 1‐year outcomes after discharge were all‐cause death, CV death, all‐cause rehospitalization, and HF rehospitalization. CV death included sudden cardiac death and deaths attributed to HF, cerebrovascular disease, coronary heart disease, or another cardiovascular cause. When no alternative nonvascular cause was evident, the death was classified as CV. Events were reported by patients or immediate family members and were not centrally adjudicated.

### 4.4 Assessments of Other Variables

Trained staff entered demographic, examination, laboratory, medical‐history, and discharge‐medication information from electronic and/or paper records into a central online database. Variables included age, sex, BMI, SBP, heart rate, pulmonary rales, peripheral edema, jugular venous distention, NYHA class, NT‐proBNP, LVEF, hypertension, atrial fibrillation (AF), COPD, anemia, and ACEI/ARB, ARNI, beta‐receptor blocker, MRA, and SGLT‐2i use. BMI was weight (kg)/height^2^ (m^2^). We defined HFrEF as LVEF <40%, HFmrEF as 40%≤ LVEF<50%, and HFpEF as LVEF ≥50%. In HFrEF, GDMT definitions depended on enrollment period. Before February 4, 2021, GDMT meant ACEI/ARB/ARNI plus a beta‐receptor blocker and MRA [32]. From February 4, 2021 onward, SGLT‐2i was added because it had been approved in China for HFrEF with or without T2D. Patients unable to take one or more classes because of documented intolerance or contraindication were considered to receive GDMT when all tolerated components were prescribed. Patients constrained to any of the core medications but prescribed at least one were also categorized as receiving GDMT.

### 4.5 Statistical Analysis

Normally distributed continuous variables are summarized as mean (standard deviation [SD]), non‐normal variables as median (IQR), and categorical variables as *n* (%). We assessed normality with the Shapiro–Wilk test; comparisons used Kruskal–Wallis tests for continuous variables and chi‐square tests for categorical variables across CKM‐count or pattern groups. Cox proportional‐hazards models estimated associations of CKM counts and patterns with outcomes, adjusting for age, sex, BMI, SBP, heart rate, NYHA class, NT‐proBNP, LVEF, hypertension, AF, COPD, anemia, and discharge medications (ACEI/ARB, ARNI, beta‐receptor blocker, MRA, SGLT‐2i). Schoenfeld residuals indicated no violation of proportional‐hazards assumptions. Fine‐Gray models treated non‐CV death as competing risk for CV death; death was a competing event for HF or all‐cause rehospitalization. We repeated analyses within LVEF and CKD‐stage strata because CKM prevalence and event rates differed across these groups.

Medication‐effect modification was evaluated in subgroups defined by GDMT, ACEI/ARB, ARNI, beta‐receptor blocker, MRA, and SGLT‐2i use (yes/no). We tested trends across CKM counts or patterns and included medication‐by‐CKM interaction terms. GDMT analyses were restricted to HFrEF; the other medication analyses were performed within each LVEF phenotype. The GDMT subgroup analysis was only conducted in patients with HFrEF, and the other subgroup analyses were conducted in each LVEF phenotype.

In this study, NT‐proBNP and BNP were used interchangeably, and 41,890 patients (33.1%) lacked NT‐proBNP values. We imputed missing NT‐proBNP using a Markov chain Monte Carlo approach and used the mean of 10 imputed values for the primary analysis; the imputation model included major baseline variables, including age, sex, BMI, SBP, heart rate, LVEF, NYHA class, medical history, and medication use. We evaluated missing‐data influence with a propensity‐based sensitivity analysis [33]. Logistic regression estimated the probability of missing NT‐proBNP from demographic, clinical, and health‐status variables; the inverse probability was used to weight observed data [34], after which the Cox models were repeated. To reduce potential reverse causation, we repeated the models after excluding patients who died within 1 month after discharge.

Analyses were conducted with SAS 9.4 (SAS Institute, Cary, NC, USA) and R 4.3.2 (R Foundation for Statistical Computing, Vienna, Austria). Two‐sided *p* < 0.05 indicated statistical significance.

## Author Contributions

Jiefu Yang and Fang Wang attest that all listed authors meet authorship criteria and that no others meeting the criteria have been omitted. All the authors were responsible for the decision to submit the manuscript. Lubi Lei has directly accessed and verified the underlying data. Concept and design: Xiang Wang, Jingkuo Li, Yi Li, Lubi Lei, Wei Wang, and Lihua Zhang. Acquisition of data: Jiefu Yang and Fang Wang. Formal analysis and interpretation of data: Lubi Lei, Jingkuo Li, Xiang Wang, Yi Li, and Wei Wang. Statistical analysis: Lubi Lei. Drafting of the manuscript: Xiang Wang, Jingkuo Li, and Lubi Lei. Critical revision of the manuscript for important intellectual content: Xiang Wang, Yi Li, Wei Wang, Xuyang Meng, Chenxi Xia, Chenguang Yang, Lihua Zhang, Yuhua Liao, Weimin Li, Yugang Dong, Xinli Li, Jingmin Zhou, Fang Wang, and Jiefu Yang. Obtained funding: Jiefu Yang. Obtained funding: Jiefu Yang. Administrative, technical, or material support: Jiefu Yang and Fang Wang. All authors have read and approved the final manuscript.

## Funding

This work was supported by the National High Level Hospital Clinical Research Funding (No. BJ‐2024‐180), National Natural Science Foundation of China (No. 82470363), and National High Level Hospital Clinical Research Funding (BJ‐2023‐170). The funders of the study have no role in study design, data collection, data analysis, data interpretation, or writing of the report.

## Ethics Statement

The study was approved by the Central Ethics Committee of Beijing Hospital (2023BJYYEC‐024‐04).

## Conflicts of Interest

The authors declare no conflicts of interest.

## Supporting information




**Supporting File**: 1 mco271008‐sup‐0001‐SuppMat.docx

## Data Availability

The datasets generated and/or analyzed during the present study are not publicly available because of government policy stipulations; it is not permissible for researchers to make the raw data publicly available at this time. Currently, it is not yet possible for other researchers to apply for access.
