# Supplementary material for: Cardiovascular–Kidney–Metabolic Overlaps, Clinical Outcomes, and Medications Among Patients With Heart Failure: Results From the Chinese Cardiovascular Association Database–Heart Failure Center Registry
Source: MedComm (2020). 2026 Sep 23;7(10):e71008. doi: 10.1002/mco2.71008 (PMC13598551; doi:10.1002/mco2.71008)
Supplement: Supplementary file 1 [file MCO2-7-e71008-s001.docx]

**Supplemental Material**

**Full title**

Cardiovascular-Kidney-Metabolic Overlaps, Clinical Outcomes, and Medications among Patients with Heart Failure: Results from The CCA Database-HF Center Registry

**Running title**

CKM in HF and Clinical Outcomes

**Authors’ names and affiliations**

Xiang Wang, MD, PhD ^1, #^; Jingkuo Li, PhD ^2, #^; Yi Li, MD, PhD ^1, #^; Lubi Lei, PhD ^2, #^; Wei Wang, PhD ^3^; Xuyang Meng, MD, PhD ^1^; Chenxi Xia, MD, PhD ^1^; Chenguang Yang, MD ^1^; Lihua Zhang, MD, PhD ^2^; Yuhua Liao, MD ^4^; Weimin Li, MD ^5^; Yugang Dong, MD ^6^, Xinli Li, MD ^7^; Jingmin Zhou, MD ^8^; Jiefu Yang, MD, PhD ^1, *^; Fang Wang, MD, PhD ^1, *^

^#^indicates joint first authors.

^*^ indicates joint corresponding authors.

^1^ Department of Cardiology, Beijing Hospital, National Center of Gerontology, Institute of Geriatric Medicine, Chinese Academy of Medical Sciences, Beijing, China

^2^ National Clinical Research Center for Cardiovascular Diseases, Fuwai Hospital, National Center for Cardiovascular Diseases, State Key Laboratory of Cardiovascular Disease, Chinese Academy of Medical Sciences and Peking Union Medical College, Beijing, China

^3^ Center for Clinical and Epidemiologic Research, Beijing Anzhen Hospital, Capital Medical University, Beijing Institute of Heart, Lung and Blood Vessel Diseases, Beijing, China.

^4^ Union Hospital, Tongji Medical College, Huazhong University of Science and Technology, Wuhan, China.

^5^ The First Affiliated Hospital of Harbin Medical University, Harbin, China.

^6^ The First Affiliated Hospital of Sun Yat-sen University, Guangzhou, China.

^7^ The First Affiliated Hospital of Nanjing Medical University, Nanjing, China.

^8^ Department of Cardiology, Shanghai Institute of Cardiovascular Diseases, Zhongshan Hospital, Fudan University, Shanghai, China.

**Corresponding author**

Prof. Fang Wang, Department of Cardiology, Beijing Hospital, National Center of Gerontology, Institute of Geriatric Medicine, Chinese Academy of Medical Sciences, Beijing, 100730, China, Email: bjh_wangfang@163.com.

Prof. Jiefu Yang, Department of Cardiology, Beijing Hospital, National Center of Gerontology, Institute of Geriatric Medicine, Chinese Academy of Medical Sciences, Beijing, 100730, China, Email: yangjiefu2011@126.com.

**Supplementary Tables**

**STROBE Statement—checklist of items that should be included in reports of observational studies**

|  | Item No. | Recommendation | Page  No. | Relevant text from manuscript |
| --- | --- | --- | --- | --- |
| **Title and abstract** | 1 | (*a*) Indicate the study’s design with a commonly used term in the title or the abstract | 3 | Abstract |
|  |  | (*b*) Provide in the abstract an informative and balanced summary of what was done and what was found | 3 | Abstract |
| Introduction | | | |  |
| Background/rationale | 2 | Explain the scientific background and rationale for the investigation being reported | 4-5 | Introduction |
| Objectives | 3 | State specific objectives, including any prespecified hypotheses | 4-5 | Introduction |
| Methods | | | |  |
| Study design | 4 | Present key elements of study design early in the paper | 20-21 | Study population |
| Setting | 5 | Describe the setting, locations, and relevant dates, including periods of recruitment, exposure, follow-up, and data collection | 20-21 | Study population |
| Participants | 6 | (*a*) *Cohort study*—Give the eligibility criteria, and the sources and methods of selection of participants. Describe methods of follow-up  *Case-control study*—Give the eligibility criteria, and the sources and methods of case ascertainment and control selection. Give the rationale for the choice of cases and controls  *Cross-sectional study*—Give the eligibility criteria, and the sources and methods of selection of participants | 20-21 | Study population |
|  |  | (*b*) *Cohort study*—For matched studies, give matching criteria and number of exposed and unexposed  *Case-control study*—For matched studies, give matching criteria and the number of controls per case | - | - |
| Variables | 7 | Clearly define all outcomes, exposures, predictors, potential confounders, and effect modifiers. Give diagnostic criteria, if applicable | 21-22 | CKM multimorbidity assessment |
| Data sources/ measurement | 8* | For each variable of interest, give sources of data and details of methods of assessment (measurement). Describe comparability of assessment methods if there is more than one group | 21-22 | Study population |
| Bias | 9 | Describe any efforts to address potential sources of bias | 23-24 | Statistical analysis |
| Study size | 10 | Explain how the study size was arrived at | 20-23 | Study population |

Continued on next page

|  | Item No. | Recommendation | Page  No. | Relevant text from manuscript |
| --- | --- | --- | --- | --- |
| Quantitative variables | 11 | Explain how quantitative variables were handled in the analyses. If applicable, describe which groupings were chosen and why | 23-24 | Statistical analysis |
| Statistical methods | 12 | (*a*) Describe all statistical methods, including those used to control for confounding | 23-24 | Statistical analysis |
|  |  | (*b*) Describe any methods used to examine subgroups and interactions | 23-24 | Statistical analysis |
|  |  | (*c*) Explain how missing data were addressed | 23-24 | Statistical analysis |
|  |  | (*d*) *Cohort study*—If applicable, explain how loss to follow-up was addressed  *Case-control study*—If applicable, explain how matching of cases and controls was addressed  *Cross-sectional study*—If applicable, describe analytical methods taking account of sampling strategy | 23-24 | Statistical analysis |
|  |  | (*e*) Describe any sensitivity analyses | 23-24 | Statistical analysis |
| Results | | | | |
| Participants | 13* | (a) Report numbers of individuals at each stage of study—eg numbers potentially eligible, examined for eligibility, confirmed eligible, included in the study, completing follow-up, and analysed | 6 | Result |
|  |  | (b) Give reasons for non-participation at each stage | 6 | Result |
|  |  | (c) Consider use of a flow diagram | 6 | Result |
| Descriptive data | 14* | (a) Give characteristics of study participants (eg demographic, clinical, social) and information on exposures and potential confounders | 6-8 | Result |
|  |  | (b) Indicate number of participants with missing data for each variable of interest | 6-8 | Result |
|  |  | (c) *Cohort study*—Summarise follow-up time (eg, average and total amount) | 6-8 | Result |
| Outcome data | 15* | *Cohort study*—Report numbers of outcome events or summary measures over time | 6-8 | Result |
|  |  | *Case-control study—*Report numbers in each exposure category, or summary measures of exposure | 6-8 | Result |
|  |  | *Cross-sectional study—*Report numbers of outcome events or summary measures | 6-8 | Result |
| Main results | 16 | (*a*) Give unadjusted estimates and, if applicable, confounder-adjusted estimates and their precision (eg, 95% confidence interval). Make clear which confounders were adjusted for and why they were included | 8-16 | Result |
|  |  | (*b*) Report category boundaries when continuous variables were categorized | 8-16 | Result |
|  |  | (*c*) If relevant, consider translating estimates of relative risk into absolute risk for a meaningful time period | 8-16 | Result |

Continued on next page

|  | Item No. | Recommendation | Page  No. | Relevant text from manuscript |
| --- | --- | --- | --- | --- |
| Other analyses | 17 | Report other analyses done—eg analyses of subgroups and interactions, and sensitivity analyses | 8-16 | Result |
| Discussion | | | | |
| Key results | 18 | Summarise key results with reference to study objectives | 16 | Discussion |
| Limitations | 19 | Discuss limitations of the study, taking into account sources of potential bias or imprecision. Discuss both direction and magnitude of any potential bias | 19-20 | Discussion |
| Interpretation | 20 | Give a cautious overall interpretation of results considering objectives, limitations, multiplicity of analyses, results from similar studies, and other relevant evidence | 16-19 | Discussion |
| Generalisability | 21 | Discuss the generalisability (external validity) of the study results | 19-20 | Discussion |
| Other information | |  | | |
| Funding | 22 | Give the source of funding and the role of the funders for the present study and, if applicable, for the original study on which the present article is based | 25 | Discussion |

*Give information separately for cases and controls in case-control studies and, if applicable, for exposed and unexposed groups in cohort and cross-sectional studies.

**Note:** An Explanation and Elaboration article discusses each checklist item and gives methodological background and published examples of transparent reporting. The STROBE checklist is best used in conjunction with this article (freely available on the Web sites of PLoS Medicine at http://www.plosmedicine.org/, Annals of Internal Medicine at http://www.annals.org/, and Epidemiology at http://www.epidem.com/). Information on the STROBE Initiative is available at www.strobe-statement.org.

**Table S1.** **Baseline characteristics by combinations of CKM conditions**

| Variables | None  (n=42,472) | ASCVD only  (n=27,387) | CKD only  (n=9966) | T2D only  (n=12,538) | ASCVD + CKD  (n=7176) | CKD + T2D  (n=5387) | ASCVD + T2D  (n=15,111) | ASCVD + CKD + T2D  (n=6487) | P value |
| --- | --- | --- | --- | --- | --- | --- | --- | --- | --- |
| Age, years (mean (SD)) | 65.40 (14.99) | 68.86 (12.29) | 72.07 (14.42) | 66.37 (12.83) | 75.39 (11.42) | 70.91 (12.12) | 67.90 (11.16) | 71.90 (10.66) | <0.001 |
| Male, n (%) | 26566 (62.5) | 19104 (69.8) | 5807 (58.3) | 7340 (58.5) | 4519 (63.0) | 2721 (50.5) | 10007 (66.2) | 3767 (58.1) | <0.001 |
| BMI, kg/m^2^ (mean (SD)) | 23.93 (4.66) | 23.81 (3.99) | 23.20 (4.42) | 25.23 (5.08) | 23.10 (4.03) | 24.52 (4.60) | 24.73 (4.04) | 24.38 (3.98) | <0.001 |
| SBP, mmHg (mean (SD)) | 125.00 (21.35) | 125.69 (21.65) | 124.18 (21.51) | 128.04 (21.34) | 125.70 (22.39) | 129.86 (21.88) | 127.67 (21.57) | 129.80 (21.87) | <0.001 |
| Heart rate, bpm (mean (SD)) | 81.62 (19.45) | 78.32 (17.39) | 77.28 (16.40) | 80.57 (17.61) | 76.54 (15.92) | 76.76 (14.88) | 78.98 (16.47) | 76.77 (15.17) | <0.001 |
| NT-proBNP, pg/ml, (mean (SD)) | 605.11 (1543.72) | 515.24 (1296.69) | 932.18 (1920.68) | 563.82 (1353.19) | 947.66 (2008.68) | 992.15 (1998.00) | 541.51 (1373.13) | 951.81 (1848.31) | <0.001 |
| LVEF phenotypes, n (%) |  |  |  |  |  |  |  |  | <0.001 |
| HFrEF | 17513 (41.2) | 8190 (29.9) | 3630 (36.4) | 4953 (39.5) | 2225 (31.0) | 1597 (29.6) | 4715 (31.2) | 1897 (29.2) |  |
| HFmrEF | 9302 (21.9) | 7922 (28.9) | 2289 (23.0) | 2744 (21.9) | 1980 (27.6) | 1289 (23.9) | 4470 (29.6) | 1806 (27.8) |  |
| HFpEF | 15657 (36.9) | 11275 (41.2) | 4047 (40.6) | 4841 (38.6) | 2971 (41.4) | 2501 (46.4) | 5926 (39.2) | 2784 (42.9) |  |
| NYHA class, n (%) |  |  |  |  |  |  |  |  | <0.001 |
| I | 11515 (27.1) | 8930 (32.6) | 1676 (16.8) | 3112 (24.8) | 1421 (19.8) | 908 (16.9) | 4449 (29.4) | 1242 (19.1) |  |
| II | 11593 (27.3) | 7829 (28.6) | 3937 (39.5) | 3771 (30.1) | 2714 (37.8) | 2086 (38.7) | 4563 (30.2) | 2392 (36.9) |  |
| III | 12403 (29.2) | 6908 (25.2) | 2843 (28.5) | 3527 (28.1) | 1915 (26.7) | 1473 (27.3) | 3795 (25.1) | 1742 (26.9) |  |
| IV | 6961 (16.4) | 3720 (13.6) | 1510 (15.2) | 2128 (17.0) | 1126 (15.7) | 920 (17.1) | 2304 (15.2) | 1111 (17.1) |  |
| Pulmonary rale, n (%) | 11546 (27.2) | 7688 (28.1) | 2162 (21.7) | 3235 (25.8) | 1942 (27.1) | 1285 (23.9) | 4299 (28.4) | 1865 (28.7) | <0.001 |
| Peripheral edema, n (%) | 9823 (23.1) | 5205 (19.0) | 1705 (17.1) | 2990 (23.8) | 1376 (19.2) | 1163 (21.6) | 3142 (20.8) | 1445 (22.3) | <0.001 |
| Jugular vein distension, n (%) | 5222 (12.3) | 2592 (9.5) | 1073 (10.8) | 1326 (10.6) | 825 (11.5) | 559 (10.4) | 1397 (9.2) | 685 (10.6) | <0.001 |
| Hypertension, n (%) | 20176 (47.5) | 15844 (57.9) | 6212 (62.3) | 8267 (65.9) | 5101 (71.1) | 4124 (76.6) | 10520 (69.6) | 5301 (81.7) | <0.001 |
| Type 2 diabetes, n (%) | 0 (0.0) | 0 (0.0) | 0 (0.0) | 11056 (88.2) | 0 (0.0) | 4662 (86.5) | 14145 (93.6) | 5992 (92.4) | <0.001 |
| Stroke, n (%) | 0 (0.0) | 6822 (24.9) | 0 (0.0) | 0 (0.0) | 2326 (32.4) | 0 (0.0) | 3791 (25.1) | 2024 (31.2) | <0.001 |
| MI, n (%) | 0 (0.0) | 16150 (59.0) | 0 (0.0) | 0 (0.0) | 3916 (54.6) | 0 (0.0) | 9532 (63.1) | 3844 (59.3) | <0.001 |
| PCI, n (%) | 0 (0.0) | 9716 (35.5) | 0 (0.0) | 0 (0.0) | 1409 (19.6) | 0 (0.0) | 5511 (36.5) | 1578 (24.3) | <0.001 |
| CABG, n (%) | 0 (0.0) | 557 (2.0) | 0 (0.0) | 0 (0.0) | 122 (1.7) | 0 (0.0) | 391 (2.6) | 156 (2.4) | <0.001 |
| Peripheral artery disease, n (%) | 0 (0.0) | 5405 (19.7) | 0 (0.0) | 0 (0.0) | 1972 (27.5) | 0 (0.0) | 2995 (19.8) | 1856 (28.6) | <0.001 |
| CKD, n (%) | 0 (0.0) | 0 (0.0) | 4833 (48.5) | 0 (0.0) | 4211 (58.7) | 3028 (56.2) | 0 (0.0) | 4252 (65.5) | <0.001 |
| AF, n (%) | 14927 (35.1) | 6651 (24.3) | 3817 (38.3) | 3776 (30.1) | 2314 (32.2) | 1520 (28.2) | 3105 (20.5) | 1494 (23.0) | <0.001 |
| COPD, n (%) | 4135 (9.7) | 2460 (9.0) | 1158 (11.6) | 937 (7.5) | 887 (12.4) | 454 (8.4) | 945 (6.3) | 512 (7.9) | <0.001 |
| Anemia, n (%) | 98 (0.2) | 58 (0.2) | 141 (1.4) | 39 (0.3) | 73 (1.0) | 103 (1.9) | 41 (0.3) | 90 (1.4) | <0.001 |
| ACEI/ARB, n (%) | 20538 (48.4) | 14754 (53.9) | 3503 (35.1) | 6039 (48.2) | 2648 (36.9) | 1785 (33.1) | 7842 (51.9) | 2332 (35.9) | <0.001 |
| ARNI, n (%) | 14885 (35.0) | 7655 (28.0) | 4062 (40.8) | 4941 (39.4) | 2457 (34.2) | 2205 (40.9) | 5067 (33.5) | 2375 (36.6) | <0.001 |
| Beta-receptor blocker, n (%) | 34163 (80.4) | 22294 (81.4) | 7684 (77.1) | 10340 (82.5) | 5480 (76.4) | 4160 (77.2) | 12743 (84.3) | 5208 (80.3) | <0.001 |
| MRA, n (%) | 33964 (80.0) | 19406 (70.9) | 7288 (73.1) | 9802 (78.2) | 4948 (69.0) | 3577 (66.4) | 10920 (72.3) | 4225 (65.1) | <0.001 |
| SGLT2i, n (%) | 1385 (3.3) | 381 (1.4) | 529 (5.3) | 861 (6.9) | 194 (2.7) | 421 (7.8) | 495 (3.3) | 276 (4.3) | <0.001 |

**Abbreviations**: ACEI: angiotensin converting enzyme inhibitors; AF: atrial fibrillation; ARB: angiotensin receptor blocker; ARNI: angiotensin receptor neprilysin inhibitor; ASCVD: atherosclerotic cardiovascular disease; BMI: body mass index; CABG: coronary artery bypass grafting; COPD: chronic obstructive pulmonary disease; HFmrEF: HF with a mildly reduced fraction; HFpEF: HF with preserved ejection fraction; HFrEF: HF with reduced ejection fraction; LVEF: left ventricular ejection fraction; MI: myocardial infarction; MRA: mineralocorticoid receptor antagonist; NT-proBNP: N-terminal pro-brain natriuretic peptide; NYHA: New York Heart Association; PCI: percutaneous coronary intervention; SBP: systolic blood pressure; SD: standard deviation; SGLT2i: sodium-glucose cotransporter 2 inhibitors.

**Table S2. Baseline characteristics by missing** **NT-proBNP or not**

| Variables | Not missing NT-proBNP  (n=84,634) | Missing NT-proBNP  (n=41,890) | P value |
| --- | --- | --- | --- |
| Age, years (mean (SD)) | 67.97 (13.59) | 68.67 (13.42) | <0.001 |
| Male, n (%) | 53506 (63.2) | 26325 (62.8) | 0.193 |
| BMI, kg/m^2^ (mean (SD)) | 24.03 (4.41) | 24.16 (4.52) | <0.001 |
| SBP, mmHg (mean (SD)) | 126.08 (21.92) | 126.44 (21.02) | 0.006 |
| Heart rate, bpm (mean (SD)) | 79.73 (18.10) | 78.75 (17.09) | <0.001 |
| LVEF phenotypes, n (%) |  |  | <0.001 |
| HFrEF | 30744 (36.3) | 13976 (33.4) |  |
| HFmrEF | 21459 (25.4) | 10343 (24.7) |  |
| HFpEF | 32431 (38.3) | 17571 (41.9) |  |
| NYHA class, n (%) |  |  | <0.001 |
| I | 22165 (26.2) | 11088 (26.5) |  |
| II | 25208 (29.8) | 13677 (32.6) |  |
| III | 23063 (27.3) | 11543 (27.6) |  |
| IV | 14198 (16.8) | 5582 (13.3) |  |
| Pulmonary rale, n (%) | 23469 (27.7) | 10553 (25.2) | <0.001 |
| Peripheral edema, n (%) | 18134 (21.4) | 8715 (20.8) | <0.001 |
| Jugular vein distension, n (%) | 9181 (10.8) | 4498 (10.7) | <0.001 |
| Hypertension, n (%) | 50126 (59.2) | 25419 (60.7) | <0.001 |
| Type 2 diabetes, n (%) | 23952 (28.3) | 11903 (28.4) | 0.676 |
| Stroke, n (%) | 10156 (12.0) | 4807 (11.5) | 0.007 |
| MI, n (%) | 22800 (26.9) | 10642 (25.4) | <0.001 |
| PCI, n (%) | 12614 (14.9) | 5600 (13.4) | <0.001 |
| CABG, n (%) | 853 (1.0) | 373 (0.9) | 0.048 |
| Peripheral artery disease, n (%) | 7925 (9.4) | 4303 (10.3) | <0.001 |
| CKD, n (%) | 10787 (12.7) | 5537 (13.2) | 0.019 |
| AF, n (%) | 25457 (30.1) | 12147 (29.0) | <0.001 |
| COPD, n (%) | 7448 (8.8) | 4040 (9.6) | <0.001 |
| Anemia, n (%) | 402 (0.5) | 241 (0.6) | 0.02 |
| ACEI/ARB, n (%) | 39186 (46.3) | 20255 (48.4) | <0.001 |
| ARNI, n (%) | 30002 (35.4) | 13645 (32.6) | <0.001 |
| Beta-receptor blocker, n (%) | 68858 (81.4) | 33214 (79.3) | <0.001 |
| MRA, n (%) | 63297 (74.8) | 30833 (73.6) | <0.001 |
| SGLT2i, n (%) | 3066 (3.6) | 1476 (3.5) | 0.381 |

**Abbreviations**: ACEI: angiotensin converting enzyme inhibitors; AF: atrial fibrillation; ARB: angiotensin receptor blocker; ARNI: angiotensin receptor neprilysin inhibitor; BMI: body mass index; CABG: coronary artery bypass grafting; CKD: chronic kidney disease; COPD: chronic obstructive pulmonary disease; MI: myocardial infarction; MRA: mineralocorticoid receptor antagonist; NT-proBNP: N-terminal pro-brain natriuretic peptide; NYHA: New York Heart Association; PCI: percutaneous coronary intervention; SBP: systolic blood pressure; SD: standard deviation; SGLT2i: sodium-glucose cotransporter 2 inhibitors.

**Table S3. Baseline characteristics by LVEF phenotypes**

| Variables | HFrEF  (n=44,720) | HFmrEF  (n=31,802) | HFpEF  (n=50,002) | P value |
| --- | --- | --- | --- | --- |
| Age, years (mean (SD)) | 63.78 (13.98) | 68.23 (13.04) | 72.14 (12.17) | <0.001 |
| Male, n (%) | 32246 (72.1) | 20780 (65.3) | 26805 (53.6) | <0.001 |
| BMI, kg/m^2^ (mean (SD)) | 24.04 (4.61) | 24.06 (4.35) | 24.11 (4.35) | 0.054 |
| SBP, mmHg (mean (SD)) | 121.20 (20.36) | 126.76 (21.43) | 130.32 (21.93) | <0.001 |
| Heart rate, bpm (mean (SD)) | 81.33 (17.91) | 79.77 (18.05) | 77.44 (17.27) | <0.001 |
| NT-proBNP, pg/ml, (mean (SD)) | 831.82 (1807.45) | 684.78 (1626.59) | 473.91 (1251.23) | <0.001 |
| NYHA class, n (%) |  |  |  | <0.001 |
| I | 10658 (23.8) | 8392 (26.4) | 14203 (28.4) |  |
| II | 11978 (26.8) | 10094 (31.7) | 16813 (33.6) |  |
| III | 12840 (28.7) | 8280 (26.0) | 13486 (27.0) |  |
| IV | 9244 (20.7) | 5036 (15.8) | 5500 (11.0) |  |
| Pulmonary rale, n (%) | 12913 (28.9) | 8663 (27.2) | 12446 (24.9) | <0.001 |
| Peripheral edema, n (%) | 9820 (22.0) | 6236 (19.6) | 10793 (21.6) | <0.001 |
| Jugular vein distension, n (%) | 5559 (12.4) | 3174 (10.0) | 4946 (9.9) | <0.001 |
| Hypertension, n (%) | 21822 (48.8) | 19173 (60.3) | 34550 (69.1) | <0.001 |
| Type 2 diabetes, n (%) | 11755 (26.3) | 9390 (29.5) | 14710 (29.4) | <0.001 |
| Stroke, n (%) | 3991 (8.9) | 3694 (11.6) | 7278 (14.6) | <0.001 |
| MI, n (%) | 10790 (24.1) | 11211 (35.3) | 11441 (22.9) | <0.001 |
| PCI, n (%) | 5274 (11.8) | 5688 (17.9) | 7252 (14.5) | <0.001 |
| CABG, n (%) | 369 (0.8) | 386 (1.2) | 471 (0.9) | <0.001 |
| Peripheral artery disease, n (%) | 3447 (7.7) | 2963 (9.3) | 5818 (11.6) | <0.001 |
| CKD, n (%) | 5325 (11.9) | 4054 (12.7) | 6945 (13.9) | <0.001 |
| AF, n (%) | 10235 (22.9) | 9422 (29.6) | 17947 (35.9) | <0.001 |
| COPD, n (%) | 3278 (7.3) | 2711 (8.5) | 5499 (11.0) | <0.001 |
| Anemia, n (%) | 130 (0.3) | 140 (0.4) | 373 (0.7) | <0.001 |
| ACEI/ARB, n (%) | 16884 (37.8) | 14846 (46.7) | 27711 (55.4) | <0.001 |
| ARNI, n (%) | 22801 (51.0) | 11376 (35.8) | 9470 (18.9) | <0.001 |
| Beta-receptor blocker, n (%) | 38709 (86.6) | 26302 (82.7) | 37061 (74.1) | <0.001 |
| MRA, n (%) | 39530 (88.4) | 23739 (74.6) | 30861 (61.7) | <0.001 |
| SGLT2i, n (%) | 2341 (5.2) | 1120 (3.5) | 1081 (2.2) | <0.001 |

**Abbreviations**: ACEI: angiotensin converting enzyme inhibitors; AF: atrial fibrillation; ARB: angiotensin receptor blocker; ARNI: angiotensin receptor neprilysin inhibitor; BMI: body mass index; CABG: coronary artery bypass grafting; CKD: chronic kidney disease; COPD: chronic obstructive pulmonary disease; MI: myocardial infarction; MRA: mineralocorticoid receptor antagonist; NT-proBNP: N-terminal pro-brain natriuretic peptide; NYHA: New York Heart Association; PCI: percutaneous coronary intervention; SBP: systolic blood pressure; SD: standard deviation; SGLT2i: sodium-glucose cotransporter 2 inhibitors.

**Table S4. Subgroup analysis of numbers of CKM conditions by CKD stage**

| Outcomes | Numbers of CKM conditions | | | |
| --- | --- | --- | --- | --- |
|  | 0 | 1 | 2 | 3 |
| 1-year all-cause death |  |  |  |  |
| CKD stage 1 (n=19502) ^a^ | Ref | 1.10 (0.88, 1.36) | 1.56 (1.20, 2.05) | 2.77 (0.99, 7.74) |
| CKD stage 2 (n=24463) | Ref | 1.23 (1.07, 1.41) | 1.54 (1.30, 1.84) | 1.29 (0.72, 2.30) |
| CKD stage 3 (n=15345) |  | Ref | 1.25 (1.09, 1.44) | 1.52 (1.28, 1.80) |
| CKD stage 4 (n=3362) |  | Ref | 1.09 (0.85, 1.38) | 1.36 (1.03, 1.79) |
| CKD stage 5 (n=849) |  | Ref | 1.17 (0.74, 1.86) | 1.08 (0.61, 1.94) |
| P for interaction | 0.650 |  |  |  |
| 1-year CV death |  |  |  |  |
| CKD stage 1 (n=19502) | Ref | 1.28 (0.91, 1.82) | 1.89 (1.24, 2.89) | 4.00 (0.96, 16.61) |
| CKD stage 2 (n=24463) | Ref | 1.40 (1.11, 1.76) | 2.33 (1.79, 3.04) | 1.59 (0.65, 3.94) |
| CKD stage 3 (n=15345) |  | Ref | 1.39 (1.11, 1.75) | 1.72 (1.30, 2.29) |
| CKD stage 4 (n=3362) |  | Ref | 1.36 (0.91, 2.04) | 1.79 (1.14, 2.81) |
| CKD stage 5 (n=849) |  | Ref | 1.62 (0.72, 3.63) | 1.63 (0.59, 4.50) |
| P for interaction | 0.999 |  |  |  |
| 1-year all-cause  rehospitalization |  |  |  |  |
| CKD stage 1 (n=19502) | Ref | 1.20 (1.03, 1.39) | 1.37 (1.13, 1.66) | 1.75 (0.72, 4.27) |
| CKD stage 2 (n=24463) | Ref | 1.08 (0.97, 1.22) | 1.16 (1.00, 1.35) | 0.81 (0.48, 1.37) |
| CKD stage 3 (n=15345) |  | Ref | 1.08 (0.94, 1.24) | 1.28 (1.08, 1.52) |
| CKD stage 4 (n=3362) |  | Ref | 1.44 (1.07, 1.93) | 1.46 (1.04, 2.04) |
| CKD stage 5 (n=849) |  | Ref | 0.98 (0.54, 1.77) | 1.03 (0.52, 2.05) |
| P for interaction | 0.664 |  |  |  |
| 1-year CV rehospitalization |  |  |  |  |
| CKD stage 1 (n=19502) | Ref | 1.35 (1.12, 1.62) | 1.47 (1.16, 1.85) | 2.18 (0.80, 5.92) |
| CKD stage 2 (n=24463) | Ref | 1.08 (0.94, 1.23) | 1.18 (0.99, 1.40) | 0.86 (0.47, 1.56) |
| CKD stage 3 (n=15345) |  | Ref | 1.06 (0.90, 1.25) | 1.27 (1.04, 1.55) |
| CKD stage 4 (n=3362) |  | Ref | 1.42 (1.00, 2.03) | 1.42 (0.95, 2.14) |
| CKD stage 5 (n=849) |  | Ref | 0.84 (0.39, 1.83) | 0.97 (0.39, 2.43) |
| P for interaction | 0.143 |  |  |  |

a CKD stages were categorized by eGFR (N=63521 with available eGFR data). Stage 1 refers to eGFR>=90; Stage 2 refers to 90>eGFR>=60; Stage 3 refers to 60>eGFR>=30; Stage 4 refers to 30>eGFR>=15; Stage 5 refers to 15>eGFR.

**Abbreviations:** CV: cardiovascular; Ref: reference.

**Table S5. Subgroup analysis of combinations of CKM conditions by CKD stage**

| Outcomes | None | ASCVD only | CKD only | T2D only | ASCVD + CKD | CKD + T2D | ASCVD + T2D | ASCVD + CKD + T2D |
| --- | --- | --- | --- | --- | --- | --- | --- | --- |
| 1-year all-cause death |  |  |  |  |  |  |  |  |
| CKD stage 1 (n=19502) ^a^ | Ref | 1.05 (0.82, 1.36) | 1.70 (0.57, 5.03) | 1.14 (0.85, 1.53) | 2.29 (0.73, 7.19) | 1.56 (0.38, 6.42) | 1.55 (1.18, 2.03) | 2.78 (1.00, 7.76) |
| CKD stage 2 (n=24463) | Ref | 1.23 (1.05, 1.44) | 1.16 (0.70, 1.92) | 1.24 (1.01, 1.51) | 2.26 (1.47, 3.49) | 1.23 (0.60, 2.49) | 1.50 (1.24, 1.80) | 1.29 (0.72, 2.30) |
| CKD stage 3 (n=15345) |  |  | Ref |  | 1.35 (1.16, 1.56) | 1.08 (0.89, 1.31) |  | 1.51 (1.27, 1.79) |
| CKD stage 4 (n=3362) |  |  | Ref |  | 1.04 (0.78, 1.39) | 1.14 (0.85, 1.52) |  | 1.36 (1.03, 1.80) |
| CKD stage 5 (n=849) |  |  | Ref |  | 1.19 (0.67, 2.12) | 1.15 (0.67, 1.98) |  | 1.08 (0.60, 1.94) |
| P for interaction | 0.446 |  |  |  |  |  |  |  |
| 1-year CV death |  |  |  |  |  |  |  |  |
| CKD stage 1 (n=19502) ^a^ | Ref | 1.18 (0.78, 1.79) | 1.62 (0.27, 9.71) | 1.44 (0.91, 2.27) | 0.00 (0.00, 0.00) | 1.85 (0.23, 14.83) | 1.94 (1.27, 2.96) | 4.02 (0.97, 16.69) |
| CKD stage 2 (n=24463) | Ref | 1.42 (1.09, 1.85) | 1.27 (0.59, 2.76) | 1.37 (0.98, 1.91) | 2.18 (1.05, 4.54) | 1.70 (0.63, 4.60) | 2.39 (1.81, 3.14) | 1.59 (0.65, 3.94) |
| CKD stage 3 (n=15345) |  |  | Ref |  | 1.49 (1.16, 1.90) | 1.22 (0.89, 1.68) |  | 1.71 (1.29, 2.28) |
| CKD stage 4 (n=3362) |  |  | Ref |  | 1.51 (0.96, 2.38) | 1.18 (0.72, 1.95) |  | 1.77 (1.12, 2.78) |
| CKD stage 5 (n=849) |  |  | Ref |  | 1.28 (0.47, 3.44) | 1.99 (0.76, 5.24) |  | 1.66 (0.59, 4.65) |
| P for interaction | 0.837 |  |  |  |  |  |  |  |
| 1-year all-cause  rehospitalization |  |  |  |  |  |  |  |  |
| CKD stage 1 (n=19502) ^a^ | Ref | 1.13 (0.94, 1.35) | 1.36 (0.62, 3.01) | 1.30 (1.07, 1.58) | 2.37 (0.96, 5.82) | 1.40 (0.52, 3.76) | 1.35 (1.11, 1.63) | 1.75 (0.72, 4.26) |
| CKD stage 2 (n=24463) | Ref | 1.12 (0.98, 1.28) | 0.90 (0.59, 1.36) | 1.06 (0.90, 1.24) | 1.20 (0.77, 1.87) | 1.27 (0.78, 2.06) | 1.15 (0.98, 1.35) | 0.81 (0.48, 1.37) |
| CKD stage 3 (n=15345) |  |  | Ref |  | 1.04 (0.88, 1.22) | 1.14 (0.95, 1.36) |  | 1.29 (1.09, 1.52) |
| CKD stage 4 (n=3362) |  |  | Ref |  | 1.52 (1.07, 2.15) | 1.37 (0.98, 1.91) |  | 1.46 (1.04, 2.04) |
| CKD stage 5 (n=849) |  |  | Ref |  | 0.75 (0.32, 1.76) | 1.13 (0.59, 2.16) |  | 1.04 (0.52, 2.06) |
| P for interaction | 0.914 |  |  |  |  |  |  |  |
| 1-year CV rehospitalization |  |  |  |  |  |  |  |  |
| CKD stage 1 (n=19502) ^a^ | Ref | 1.30 (1.05, 1.61) | 1.41 (0.53, 3.74) | 1.41 (1.12, 1.78) | 2.20 (0.70, 6.95) | 1.66 (0.53, 5.18) | 1.44 (1.14, 1.83) | 2.18 (0.80, 5.92) |
| CKD stage 2 (n=24463) | Ref | 1.09 (0.93, 1.27) | 1.11 (0.72, 1.73) | 1.05 (0.87, 1.27) | 1.13 (0.66, 1.92) | 1.44 (0.84, 2.47) | 1.16 (0.97, 1.39) | 0.86 (0.47, 1.56) |
| CKD stage 3 (n=15345) |  |  | Ref |  | 1.10 (0.91, 1.33) | 0.99 (0.80, 1.23) |  | 1.27 (1.04, 1.54) |
| CKD stage 4 (n=3362) |  |  | Ref |  | 1.57 (1.04, 2.36) | 1.29 (0.86, 1.95) |  | 1.42 (0.95, 2.12) |
| CKD stage 5 (n=849) |  |  | Ref |  | 0.56 (0.17, 1.84) | 1.04 (0.46, 2.38) |  | 0.97 (0.39, 2.45) |
| P for interaction | 0.288 |  |  |  |  |  |  |  |

a CKD stages were categorized by eGFR (N=63521 with available eGFR data). Stage 1 refers to eGFR>=90; Stage 2 refers to 90>eGFR>=60; Stage 3 refers to 60>eGFR>=30; Stage 4 refers to 30>eGFR>=15; Stage 5 refers to 15>eGFR.

**Abbreviations:** CV: cardiovascular; Ref: reference.

**Table S6. Sensitivity analysis of the numbers of CKM conditions**

| Outcomes | Numbers of CKM conditions | | | |
| --- | --- | --- | --- | --- |
|  | 0 | 1 | 2 | 3 |
| 1-year all-cause death |  |  |  |  |
| Main analysis | 1463 (3.44) | 2239 (4.49) | 1699 (6.14) | 560 (8.63) |
|  | Ref | 1.29 (1.21, 1.38) | 1.76 (1.64, 1.89) | 2.43 (2.20, 2.69) |
|  | Ref | <0.001 | <0.001 | <0.001 |
| Excluding participants died within 30 days after discharge | 1123 (2.67) | 1636 (3.32) | 1258 (4.62) | 405 (6.40) |
|  | Ref | 1.22 (1.13, 1.31) | 1.69 (1.55, 1.83) | 2.30 (2.04, 2.59) |
|  | Ref | <0.001 | <0.001 | <0.001 |
| Adjusting for missing NT-proBNP by IPTW | 1463 (3.44) | 2242 (4.50) | 1707 (6.17) | 561 (8.65) |
|  | Ref | 1.29 (1.21, 1.38) | 1.76 (1.64, 1.90) | 2.43 (2.19, 2.69) |
|  | Ref | <0.001 | <0.001 | <0.001 |
| 1-year CV death |  |  |  |  |
| Main analysis | 588 (1.38) | 948 (1.90) | 756 (2.73) | 255 (3.93) |
|  | Ref | 1.40 (1.27, 1.56) | 2.04 (1.82, 2.28) | 2.93 (2.51, 3.41) |
|  | Ref | <0.001 | <0.001 | <0.001 |
| Excluding participants died within 30 days after discharge | 433 (1.03) | 672 (1.36) | 532 (1.95) | 174 (2.75) |
|  | Ref | 1.35 (1.20, 1.53) | 1.97 (1.73, 2.25) | 2.80 (2.33, 3.36) |
|  | Ref | <0.001 | <0.001 | <0.001 |
| Adjusting for missing NT-proBNP by IPTW | 587 (1.38) | 947 (1.90) | 760 (2.74) | 256 (3.95) |
|  | Ref | 1.40 (1.26, 1.55) | 2.04 (1.83, 2.28) | 2.93 (2.51, 3.42) |
|  | Ref | <0.001 | <0.001 | <0.001 |
| 1-year all-cause  rehospitalization |  |  |  |  |
| Main analysis | 2357 (5.55) | 3085 (6.18) | 1966 (7.10) | 566 (8.73) |
|  | Ref | 1.13 (1.07, 1.20) | 1.31 (1.24, 1.40) | 1.63 (1.48, 1.79) |
|  | Ref | <0.001 | <0.001 | <0.001 |
| Excluding participants died within 30 days after discharge | 2339 (5.55) | 3065 (6.22) | 1952 (7.17) | 558 (8.81) |
|  | Ref | 1.14 (1.08, 1.20) | 1.33 (1.25, 1.41) | 1.64 (1.49, 1.80) |
|  | Ref | <0.001 | <0.001 | <0.001 |
| Adjusting for missing NT-proBNP by IPTW | 2364 (5.57) | 3085 (6.19) | 1970 (7.11) | 567 (8.74) |
|  | Ref | 1.13 (1.07, 1.19) | 1.31 (1.23, 1.39) | 1.62 (1.48, 1.78) |
|  | Ref | <0.001 | <0.001 | <0.001 |
| 1-year CV rehospitalization |  |  |  |  |
| Main analysis | 1719 (4.05) | 2240 (4.49) | 1433 (5.18) | 398 (6.14) |
|  | Ref | 1.14 (1.07, 1.21) | 1.33 (1.24, 1.43) | 1.60 (1.43, 1.79) |
|  | Ref | <0.001 | <0.001 | <0.001 |
| Excluding participants died within 30 days after discharge | 1707 (4.05) | 2223 (4.51) | 1423 (5.23) | 395 (6.24) |
|  | Ref | 1.14 (1.07, 1.22) | 1.34 (1.25, 1.44) | 1.62 (1.45, 1.82) |
|  | Ref | <0.001 | <0.001 | <0.001 |
| Adjusting for missing NT-proBNP by IPTW | 1722 (4.06) | 2239 (4.49) | 1437 (5.19) | 400 (6.17) |
|  | Ref | 1.13 (1.06, 1.21) | 1.33 (1.24, 1.43) | 1.60 (1.43, 1.79) |
|  | Ref | <0.001 | <0.001 | <0.001 |

**Abbreviations:** CV: cardiovascular; IPTW: inverse probability of treatment weighting; NT-proBNP: N-terminal pro-brain natriuretic peptide; Ref: reference.

**Table S7. Sensitivity analysis of the combinations of CKM conditions**

| Outcomes | None | ASCVD only | CKD only | T2D only | ASCVD + CKD | CKD + T2D | ASCVD + T2D | ASCVD + CKD + T2D |
| --- | --- | --- | --- | --- | --- | --- | --- | --- |
| 1-year all-cause death |  |  |  |  |  |  |  |  |
| Main analysis | 1463 (3.44) | 1083 (3.95) | 703 (7.05) | 453 (3.61) | 665 (9.27) | 356 (6.61) | 678 (4.49) | 560 (8.63) |
|  | Ref | 1.16 (1.07, 1.26) | 1.67 (1.52, 1.83) | 1.21 (1.09, 1.34) | 2.07 (1.88, 2.27) | 1.92 (1.71, 2.17) | 1.50 (1.37, 1.65) | 2.46 (2.22, 2.72) |
|  | Ref | <0.001 | <0.001 | 0.001 | <0.001 | <0.001 | <0.001 | <0.001 |
| Excluding participants died within 30 days after discharge | 1123 (2.67) | 777 (2.87) | 528 (5.39) | 331 (2.67) | 494 (7.05) | 281 (5.29) | 483 (3.24) | 405 (6.40) |
|  | Ref | 1.07 (0.98, 1.18) | 1.64 (1.47, 1.82) | 1.14 (1.00, 1.29) | 2.02 (1.81, 2.25) | 1.98 (1.73, 2.26) | 1.37 (1.23, 1.53) | 2.33 (2.07, 2.62) |
|  | Ref | 0.146 | <0.001 | 0.043 | <0.001 | <0.001 | <0.001 | <0.001 |
| Adjusting for missing NT-proBNP by IPTW | 1463 (3.44) | 1087 (3.96) | 702 (7.06) | 454 (3.62) | 668 (9.31) | 357 (6.64) | 682 (4.51) | 561 (8.65) |
|  | Ref | 1.16 (1.07, 1.26) | 1.67 (1.52, 1.83) | 1.21 (1.09, 1.34) | 2.07 (1.88, 2.28) | 1.93 (1.72, 2.17) | 1.50 (1.37, 1.65) | 2.46 (2.22, 2.72) |
|  | Ref | <0.001 | <0.001 | <0.001 | <0.001 | <0.001 | <0.001 | <0.001 |
| 1-year CV death |  |  |  |  |  |  |  |  |
| Main analysis | 588 (1.38) | 485 (1.77) | 271 (2.72) | 192 (1.53) | 282 (3.93) | 144 (2.67) | 330 (2.18) | 255 (3.93) |
|  | Ref | 1.32 (1.17, 1.49) | 1.68 (1.45, 1.95) | 1.31 (1.12, 1.55) | 2.31 (1.99, 2.67) | 2.08 (1.73, 2.50) | 1.86 (1.62, 2.14) | 2.95 (2.53, 3.44) |
|  | Ref | <0.001 | <0.001 | 0.001 | <0.001 | <0.001 | <0.001 | <0.001 |
| Excluding participants died within 30 days after discharge | 433 (1.03) | 326 (1.20) | 205 (2.09) | 141 (1.14) | 200 (2.86) | 112 (2.11) | 220 (1.47) | 174 (2.75) |
|  | Ref | 1.21 (1.04, 1.40) | 1.77 (1.49, 2.09) | 1.30 (1.07, 1.58) | 2.30 (1.94, 2.74) | 2.26 (1.83, 2.79) | 1.68 (1.42, 1.98) | 2.84 (2.36, 3.41) |
|  | Ref | 0.012 | <0.001 | 0.007 | <0.001 | <0.001 | <0.001 | <0.001 |
| Adjusting for missing NT-proBNP by IPTW | 587 (1.38) | 484 (1.77) | 270 (2.72) | 192 (1.53) | 284 (3.96) | 144 (2.68) | 331 (2.19) | 256 (3.95) |
|  | Ref | 1.32 (1.17, 1.49) | 1.68 (1.45, 1.95) | 1.32 (1.12, 1.55) | 2.32 (2.00, 2.69) | 2.08 (1.73, 2.51) | 1.86 (1.62, 2.14) | 2.95 (2.53, 3.44) |
|  | Ref | <0.001 | <0.001 | 0.001 | <0.001 | <0.001 | <0.001 | <0.001 |
| 1-year all-cause  rehospitalization |  |  |  |  |  |  |  |  |
| Main analysis | 2357 (5.55) | 1604 (5.86) | 680 (6.82) | 801 (6.39) | 562 (7.83) | 431 (8.00) | 973 (6.44) | 566 (8.73) |
|  | Ref | 1.08 (1.01, 1.15) | 1.21 (1.11, 1.32) | 1.19 (1.10, 1.29) | 1.39 (1.26, 1.53) | 1.49 (1.34, 1.66) | 1.22 (1.13, 1.31) | 1.64 (1.49, 1.80) |
|  | Ref | 0.025 | <0.001 | <0.001 | <0.001 | <0.001 | <0.001 | <0.001 |
| Excluding participants died within 30 days after discharge | 2339 (5.55) | 1595 (5.89) | 671 (6.85) | 799 (6.44) | 558 (7.97) | 428 (8.06) | 966 (6.48) | 558 (8.81) |
|  | Ref | 1.08 (1.02, 1.16) | 1.22 (1.11, 1.33) | 1.20 (1.11, 1.30) | 1.41 (1.28, 1.55) | 1.50 (1.35, 1.66) | 1.23 (1.14, 1.32) | 1.65 (1.50, 1.81) |
|  | Ref | 0.015 | <0.001 | <0.001 | <0.001 | <0.001 | <0.001 | <0.001 |
| Adjusting for missing NT-proBNP by IPTW | 2364 (5.57) | 1608 (5.87) | 678 (6.81) | 800 (6.39) | 563 (7.85) | 432 (8.02) | 975 (6.44) | 567 (8.74) |
|  | Ref | 1.07 (1.01, 1.14) | 1.21 (1.11, 1.32) | 1.19 (1.10, 1.29) | 1.39 (1.26, 1.52) | 1.49 (1.34, 1.65) | 1.21 (1.13, 1.31) | 1.63 (1.49, 1.79) |
|  | Ref | 0.029 | <0.001 | <0.001 | <0.001 | <0.001 | <0.001 | <0.001 |
| 1-year CV rehospitalization |  |  |  |  |  |  |  |  |
| Main analysis | 1719 (4.05) | 1146 (4.18) | 513 (5.15) | 581 (4.63) | 416 (5.80) | 294 (5.46) | 723 (4.78) | 398 (6.14) |
|  | Ref | 1.07 (0.99, 1.15) | 1.26 (1.14, 1.39) | 1.20 (1.09, 1.32) | 1.41 (1.27, 1.58) | 1.43 (1.26, 1.62) | 1.26 (1.16, 1.38) | 1.61 (1.44, 1.80) |
|  | Ref | 0.100 | <0.001 | <0.001 | <0.001 | <0.001 | <0.001 | <0.001 |
| Excluding participants died within 30 days after discharge | 1707 (4.05) | 1137 (4.20) | 505 (5.16) | 581 (4.68) | 413 (5.90) | 292 (5.50) | 718 (4.81) | 395 (6.24) |
|  | Ref | 1.07 (0.99, 1.15) | 1.25 (1.13, 1.39) | 1.21 (1.10, 1.33) | 1.43 (1.28, 1.60) | 1.43 (1.26, 1.62) | 1.27 (1.16, 1.39) | 1.63 (1.46, 1.83) |
|  | Ref | 0.085 | <0.001 | <0.001 | <0.001 | <0.001 | <0.001 | <0.001 |
| Adjusting for missing NT-proBNP by IPTW | 1722 (4.06) | 1148 (4.19) | 512 (5.15) | 579 (4.63) | 417 (5.81) | 295 (5.48) | 724 (4.79) | 400 (6.17) |
|  | Ref | 1.06 (0.99, 1.15) | 1.25 (1.13, 1.38) | 1.19 (1.09, 1.31) | 1.41 (1.27, 1.58) | 1.43 (1.26, 1.62) | 1.26 (1.15, 1.37) | 1.61 (1.44, 1.80) |
|  | Ref | 0.114 | <0.001 | <0.001 | <0.001 | <0.001 | <0.001 | <0.001 |

**Abbreviations:** ASCVD: atherosclerotic cardiovascular disease; CKD: chronic kidney disease; CV: cardiovascular; IPTW: inverse probability of treatment weighting; NT-proBNP: N-terminal pro-brain natriuretic peptide; Ref: reference; T2D: type 2 diabetes.

**Table S8. The proportion of GDMT use by numbers of CKM conditions in patients with HFrEF**

|  | 0  (n=17,513) | 1  (n=16,773) | 2  (n=8537) | 3  (n=1897) |
| --- | --- | --- | --- | --- |
| GDMT status |  |  |  |  |
| Complete GDMT use | 9651 (55.11) | 8381 (49.97) | 3,910 (45.80) | 685 (36.11) |
| Receiving all four components ^a^ | 707/5167 (13.68) | 734/5448 (13.47) | 344/2939 (11.70) | 85/766 (11.10) |
| Documented intolerance/contraindication to 1 GDMT component | 1111 (6.34) | 1269 (7.57) | 719 (8.42) | 209 (11.02) |
| Documented intolerance/contraindication to ≥2 GDMT components | 198 (1.13) | 252 (1.50) | 243 (2.85) | 104 (5.48) |
| Not receiving GDMT | 6553 (37.42) | 6871 (40.96) | 3665 (42.93) | 899 (47.39) |

a The proportion was calculated in patients enrolled after 4^th^ February 2021.

**Abbreviations:** GDMT: guideline-directed medical treatment.

**Table S9. The proportion of GDMT use by combinations of CKM conditions in patients with HFrEF**

|  | None  (n=17,513) | ASCVD only  (n=8190) | CKD only  (n=3630) | T2D only  (n=4953) | ASCVD + CKD  (n=2225) | CKD + T2D  (n=1597) | ASCVD + T2D  (n=4715) | ASCVD + CKD + T2D  (n=1897) |
| --- | --- | --- | --- | --- | --- | --- | --- | --- |
| GDMT status |  |  |  |  |  |  |  |  |
| Complete GDMT use | 9651 (55.11) | 4342 (53.02) | 1303 (35.90) | 2736 (55.24) | 752 (33.80) | 562 (35.19) | 2596 (55.06) | 685 (36.11) |
| Receiving all four components ^a^ | 707/5169 (13.68) | 145/1819  (7.97) | 232/1862 (12.46) | 357/1767 (20.20) | 63/932  (6.76) | 125/807  (15.49) | 156/1200 (13.00) | 85/766  (11.10) |
| Documented intolerance/contraindication to 1 GDMT component | 1111 (6.34) | 707 (8.63) | 299 (8.24) | 263 (5.31) | 232 (10.43) | 126 (7.89) | 361 (7.66) | 209 (11.02) |
| Documented intolerance/contraindication to ≥2 GDMT components | 198 (1.13) | 117 (1.43) | 94 (2.59) | 41 (0.83) | 102 (4.58) | 83 (5.20) | 58 (1.23) | 104 (5.48) |
| Not receiving GDMT | 6553 (37.42) | 3024 (36.92) | 1934 (53.28) | 1913 (38.62) | 1139 (51.19) | 826 (51.72) | 1700 (36.06) | 899 (47.39) |

a The proportion was calculated in patients enrolled after 4^th^ February 2021.

**Abbreviations:** ASCVD: atherosclerotic cardiovascular disease; CKD: chronic kidney disease; GDMT: guideline-directed medical treatment.

**Supplementary Figures:**

**Figure S1. 1-year CV death risk according to combinations of CKM conditions and LVEF phenotypes.**

(A) Univariable and multivariable analysis of CV death risk according to combinations of CKM conditions. The reference (Ref) is no CKM (B) multivariable analysis of CV death risk by combinations of CKM conditions and LVEF phenotypes.

Multivariable analysis was adjusted for age, sex, BMI, SBP, heart rate, NYHA class, NT-proBNP, LVEF, hypertension, AF, COPD, anemia, and medications prescribed at discharge (ACEI/ ARB, ARNI, beta-receptor blocker, MRA, SGLT-2i).

**Abbreviations:** ASCVD: atherosclerotic cardiovascular disease; CI: confidence interval; CKD: chronic kidney disease; CKM: cardiovascular-kidney-metabolic disease; CV: cardiovascular; HR: hazard ratio; Ref: reference; T2D: type 2 diabetes.

**Figure S2. 1-year all-cause rehospitalization risk according to combinations of CKM conditions and LVEF phenotypes.**

(A) Univariable and multivariable analysis of all-cause rehospitalization risk according to combinations of CKM conditions. The reference (Ref) is no CKM (B) multivariable analysis of all-cause rehospitalization risk by combinations of CKM conditions and LVEF phenotypes.

Multivariable analysis was adjusted for age, sex, BMI, SBP, heart rate, NYHA class, NT-proBNP, LVEF, hypertension, AF, COPD, anemia, and medications prescribed at discharge (ACEI/ ARB, ARNI, beta-receptor blocker, MRA, SGLT-2i).

**Abbreviations:** ASCVD: atherosclerotic cardiovascular disease; CI: confidence interval; CKD: chronic kidney disease; CKM: cardiovascular-kidney-metabolic disease; HR: hazard ratio; Ref: reference; T2D: type 2 diabetes.

**Figure S3. 1-year CV rehospitalization risk according to combinations of CKM conditions and LVEF phenotypes.**

(A) Univariable and multivariable analysis of CV rehospitalization risk according to combinations of CKM conditions. The reference (Ref) is no CKM (B) multivariable analysis of CV rehospitalization risk by combinations of CKM conditions and LVEF phenotypes.

Multivariable analysis was adjusted for age, sex, BMI, SBP, heart rate, NYHA class, NT-proBNP, LVEF, hypertension, AF, COPD, anemia, and medications prescribed at discharge (ACEI/ ARB, ARNI, beta-receptor blocker, MRA, SGLT-2i).

**Abbreviations:** ASCVD: atherosclerotic cardiovascular disease; CI: confidence interval; CKD: chronic kidney disease; CKM: cardiovascular-kidney-metabolic disease; CV: cardiovascular; HR: hazard ratio; Ref: reference; T2D: type 2 diabetes.

**Figure S4. Prescription rates of heart failure medications by numbers of CKM conditions at discharge**

(A) The prescription of guideline directed medical treatment in patients with HFrEF according to the numbers of CKM conditions. (B) The prescription of ACEI or ARB in patients with HFrEF, HFmrEF, and HFpEF according to the numbers of CKM conditions. (C) The prescription of ARNI in patients with HFrEF, HFmrEF, and HFpEF according to the numbers of CKM conditions. (D) The prescription of beta-receptor blockers in patients with HFrEF, HFmrEF, and HFpEF according to the numbers of CKM conditions. (E) The prescription of MRA in patients with HFrEF, HFmrEF, and HFpEF according to the numbers of CKM conditions. (F) The prescription of SGLT2i in patients with HFrEF, HFmrEF, and HFpEF according to the numbers of CKM conditions.

**Abbreviations:** ACEI: angiotensin converting enzyme inhibitors; ARB: angiotensin receptor blocker; ARNI: angiotensin receptor neprilysin inhibitor; CKM: cardiovascular-kidney-metabolic disease; MRA: mineralocorticoid receptor antagonist; SGLT2i: sodium-glucose cotransporter 2 inhibitors.

**Figure S5. 1-year risk of death and rehospitalization according to numbers of CKM conditions and ACEI/ARB treatment**

Models were adjusted for age, sex, BMI, SBP, heart rate, NYHA class, NT-proBNP, LVEF, hypertension, AF, COPD, anemia, and medications prescribed at discharge (ARNI, beta-receptor blocker, MRA, SGLT-2i).

**Abbreviations**: CI: confidence interval; CV: cardiovascular; HR: hazard ratio; Ref: reference.

**Figure S6. 1-year risk of death and rehospitalization according to numbers of CKM conditions and ARNI treatment**

Models were adjusted for age, sex, BMI, SBP, heart rate, NYHA class, NT-proBNP, LVEF, hypertension, AF, COPD, anemia, and medications prescribed at discharge (ACEI/ARB, beta-receptor blocker, MRA, SGLT-2i).

**Abbreviations**: CI: confidence interval; CV: cardiovascular; HR: hazard ratio; Ref: reference.

**Figure S7. 1-year risk of death and rehospitalization according to numbers of CKM conditions and beta-receptor blocker treatment**

Models were adjusted for age, sex, BMI, SBP, heart rate, NYHA class, NT-proBNP, LVEF, hypertension, AF, COPD, anemia, and medications prescribed at discharge (ACEI/ARB, ARNI, MRA, SGLT-2i).

**Abbreviations**: CI: confidence interval; CV: cardiovascular; HR: hazard ratio; Ref: reference.

**Figure S8. 1-year risk of death and rehospitalization according to numbers of CKM conditions and MRA treatment**

Models were adjusted for age, sex, BMI, SBP, heart rate, NYHA class, NT-proBNP, LVEF, hypertension, AF, COPD, anemia, and medications prescribed at discharge (ACEI/ARB, ARNI, beta-receptor, SGLT-2i).

**Abbreviations**: CI: confidence interval; CV: cardiovascular; HR: hazard ratio; Ref: reference

**Figure S9. 1-year risk of death and rehospitalization according to numbers of CKM conditions and SGLT-2i treatment**

Models were adjusted for age, sex, BMI, SBP, heart rate, NYHA class, NT-proBNP, LVEF, hypertension, AF, COPD, anemia, and medications prescribed at discharge (ACEI/ARB, ARNI, beta-receptor, MRA).

**Abbreviations**: CI: confidence interval; CV: cardiovascular; HR: hazard ratio; Ref: reference.

**Figure S10. Prescription rates of heart failure medications by combinations of CKM conditions at discharge**

(A) The prescription of guideline directed medical treatment in patients with HFrEF according to the combinations of CKM conditions. (B) The prescription of ACEI or ARB in patients with HFrEF, HFmrEF, and HFpEF according to the combinations of CKM conditions. (C) The prescription of ARNI in patients with HFrEF, HFmrEF, and HFpEF according to the combinations of CKM conditions. (D) The prescription of beta-receptor blockers in patients with HFrEF, HFmrEF, and HFpEF according to the combinations of CKM conditions. (E) The prescription of MRA in patients with HFrEF, HFmrEF, and HFpEF according to the combinations of CKM conditions. (F) The prescription of SGLT2i in patients with HFrEF, HFmrEF, and HFpEF according to the combinations of CKM conditions.

**Abbreviations:** ACEI: angiotensin converting enzyme inhibitors; ARB: angiotensin receptor blocker; ARNI: angiotensin receptor neprilysin inhibitor; CKM: cardiovascular-kidney-metabolic disease; MRA: mineralocorticoid receptor antagonist; SGLT2i: sodium-glucose cotransporter 2 inhibitors.

**Figure S11. 1-year risk of death and rehospitalization according to combinations of CKM conditions and GDMT treatment in patients with HFrEF**

Models were adjusted for age, sex, BMI, SBP, heart rate, NYHA class, NT-proBNP, LVEF, hypertension, AF, COPD, and anemia.

**Abbreviations**: CI: confidence interval; CV: cardiovascular; HR: hazard ratio; Ref: reference.

**Figure S12. 1-year risk of death and rehospitalization according to combinations of CKM conditions and ACEI/ARB treatment**

Models were adjusted for age, sex, BMI, SBP, heart rate, NYHA class, NT-proBNP, LVEF, hypertension, AF, COPD, anemia, and medications prescribed at discharge (ARNI, beta-receptor blocker, MRA, SGLT-2i).

**Abbreviations**: CI: confidence interval; CV: cardiovascular; HR: hazard ratio; Ref: reference.

**Figure S13. 1-year risk of death and rehospitalization according to numbers of CKM conditions and ARNI treatment**

Models were adjusted for age, sex, BMI, SBP, heart rate, NYHA class, NT-proBNP, LVEF, hypertension, AF, COPD, anemia, and medications prescribed at discharge (ACEI/ARB, beta-receptor blocker, MRA, SGLT-2i).

**Abbreviations**: CI: confidence interval; CV: cardiovascular; HR: hazard ratio; Ref: reference.

**Figure S14. 1-year risk of death and rehospitalization according to combinations of CKM conditions and beta-receptor blocker treatment**

Models were adjusted for age, sex, BMI, SBP, heart rate, NYHA class, NT-proBNP, LVEF, hypertension, AF, COPD, anemia, and medications prescribed at discharge (ACEI/ARB, ARNI, MRA, SGLT-2i).

**Abbreviations**: CI: confidence interval; CV: cardiovascular; HR: hazard ratio; Ref: reference.

**Figure S15. 1-year risk of death and rehospitalization according to combinations of CKM conditions and MRA treatment**

Models were adjusted for age, sex, BMI, SBP, heart rate, NYHA class, NT-proBNP, LVEF, hypertension, AF, COPD, anemia, and medications prescribed at discharge (ACEI/ARB, ARNI, beta-receptor, SGLT-2i).

**Abbreviations**: CI: confidence interval; CV: cardiovascular; HR: hazard ratio; Ref: reference

**Figure S16. 1-year risk of death and rehospitalization according to combinations of CKM conditions and SGLT-2i treatment**

Models were adjusted for age, sex, BMI, SBP, heart rate, NYHA class, NT-proBNP, LVEF, hypertension, AF, COPD, anemia, and medications prescribed at discharge (ACEI/ARB, ARNI, beta-receptor, MRA).

**Abbreviations**: CI: confidence interval; CV: cardiovascular; HR: hazard ratio; Ref: reference.

**Figure S17. The flow chart of participants through the study.**

**Abbreviations**: CCA: China Cardiovascular Association; HF: heart failure; LVEF: left ventricular ejection fraction.
